# Coagulase-negative staphylococci release a purine analog that inhibits *Staphylococcus aureus* virulence

**DOI:** 10.1038/s41467-021-22175-3

**Published:** 2021-03-25

**Authors:** Denny Chin, Mariya I. Goncheva, Ronald S. Flannagan, Shayna R. Deecker, Veronica Guariglia-Oropeza, Alexander W. Ensminger, David E. Heinrichs

**Affiliations:** 1 Department of Microbiology and Immunology, University of Western Ontario, London, ON Canada; 2 Department of Biochemistry, MaRS Centre, Toronto, ON Canada; 3 Department of Molecular Genetics, MaRS Centre, Toronto, ON Canada

**Keywords:** Bacterial pathogenesis, Bacterial toxins, Microbial communities, Pathogens

## Abstract

Coagulase-negative staphylococci and *Staphylococcus aureus* colonize similar niches in mammals and conceivably compete for space and nutrients. Here, we report that a coagulase-negative staphylococcus, *Staphylococcus chromogenes* ATCC43764, synthesizes and secretes 6-thioguanine (6-TG), a purine analog that suppresses *S. aureus* growth by inhibiting de novo purine biosynthesis. We identify a 6-TG biosynthetic gene cluster in *S. chromogenes* and other coagulase-negative staphylococci including *S. epidermidis*, *S. pseudintermedius* and *S. capitis*. Recombinant *S. aureus* strains harbouring this operon produce 6-TG and, when used in subcutaneous co-infections in mice with virulent *S. aureus* USA300, protect the host from necrotic lesion formation. Used prophylactically, 6-TG reduces necrotic skin lesions in mice infected with USA300, and this effect is mediated by abrogation of toxin production. RNAseq analyses reveal that 6-TG downregulates expression of genes coding for purine biosynthesis, the accessory gene regulator (agr) and ribosomal proteins in *S. aureus*, providing an explanation for its effect on toxin production.

## Abstract (web summary)

Coagulase-negative staphylococci and *Staphylococcus aureus* colonize similar niches in mammals. Here, Chin et al. show that a coagulase-negative staphylococcus secretes 6-thioguanine, a purine analog that suppresses *S. aureus* growth and virulence by inhibiting de novo purine biosynthesis and toxin production.

## Introduction

*Staphylococcus aureus* is an opportunistic human pathogen and a significant cause of morbidity and mortality worldwide, causing a wide spectrum of diseases ranging from superficial skin and soft tissue infections to severe life-threatening diseases such as endocarditis and pneumonia^1,2^. The enormous health burden caused by *S. aureus* is, in part, attributed to its ability to acquire resistance against several classes of antibiotics, making it refractory to many treatment options. Moreover, methicillin-resistant *S. aureus* (MRSA) can infect both healthy and immunocompromised populations in the community and hospital setting^3–5^. Due to the emergence of MRSA, drug discovery is critically important for combating *S. aureus* infections^6,7^.

To identify compounds that antagonize *S. aureus*, several groups have focused on coagulase-negative staphylococcal species (CoNS) as many of these species often colonize the same niches as *S. aureus* and likely compete for space and nutrients^8–10^. To successfully compete with *S. aureus*, CoNS have been shown to secrete antimicrobials that directly kill *S. aureus*^10,11^. Indeed, strains of *S. epidermidis* and *S. hominis* species, among others, have been reported to secrete posttranslationally modified peptides termed bacteriocins that inhibit *S. aureus* colonization on human skin^11^. In addition, *S. lugdunensis* has recently been shown to produce the non-ribosomally synthesized cyclic peptide antibiotic termed lugdunin that directly kills *S. aureus*^10^. Moreover, lugdunin can stimulate host production of antimicrobial peptides and recruit immune cells to clear *S. aureus* infections^12^. Another strategy used by some CoNS including *S. schleiferi* and *S. caprae* is the secretion of autoinducing peptides that specifically block the activation of the *agr* quorum sensing system in *S. aureus*. By doing so, these CoNS reduce *S. aureus* virulence and interfere with colonization of the host^9,13,14^. Clearly, CoNS have evolved mechanisms to antagonize *S. aureus* growth and virulence to gain a competitive advantage.

*S. chromogenes* is a CoNS that can secrete antimicrobials to antagonize *S. aureus* and other bacteria^8,15–17^. Here, we show that *S. chromogenes* ATCC43764 secretes the purine analog 6-thioguanine (6-TG) to impede *S. aureus* growth. The underlying genetic basis for 6-TG biosynthesis was elucidated in *S. chromogenes* as a six-gene operon, termed *tgs* for thioguanine synthesis; the *tgs* operon was also identified in the genomes of a number of *S. capitis*, *S. pseudintermedius*, and *S. epidermidis* strains. We show that the operon from *tgs*-positive CoNS strains encodes 6-TG, which is inhibitory to *S. aureus*. This was shown to be the case in vivo as well, as co-infections of virulent *S. aureus* USA300 with *tgs*-positive *S. epidermidis* or *S. aureus* RN4220 (a cloning strain) carrying *tgs* genes on a plasmid demonstrated a significant reduction in skin lesion necrosis. Moreover, 6-TG reduced *S. aureus* disease severity in a subcutaneous murine infection model, owing to its effect on downregulating toxin production by *S. aureus*. The therapeutic effects of 6-TG are due to its ability to reduce gene expression of the *agr* quorum sensing system, ribosomal proteins, and purine biosynthesis. This study identifies a mechanism by which CoNS interfere with growth and virulence of *S. aureus*.

## Results

### *S. chromogenes* ATCC43764 inhibits *S. aureus* by secreting 6-TG

**Fig. 1:**
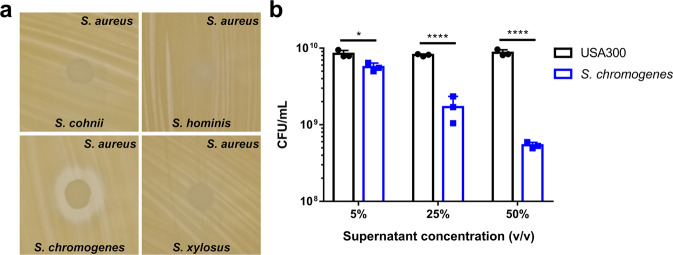
*S. chromogenes* secretes a molecule that inhibits *S. aureus* growth. **a** Simultaneous antagonism assay of selected representative CoNS on TSA. **b** *S. aureus* was cultured in TSB containing spent culture supernatant from *S. chromogenes* or *S. aureus*. The bacteria were grown for 24 h before they were plated and CFU/mL were enumerated. Results are pooled from three independent experiments. Data are shown as mean ± SD (*n* = 3, *p ≤ 0.05, ****p ≤ 0.0001. Two-sided unpaired *t*-test).

**Fig. 2:**
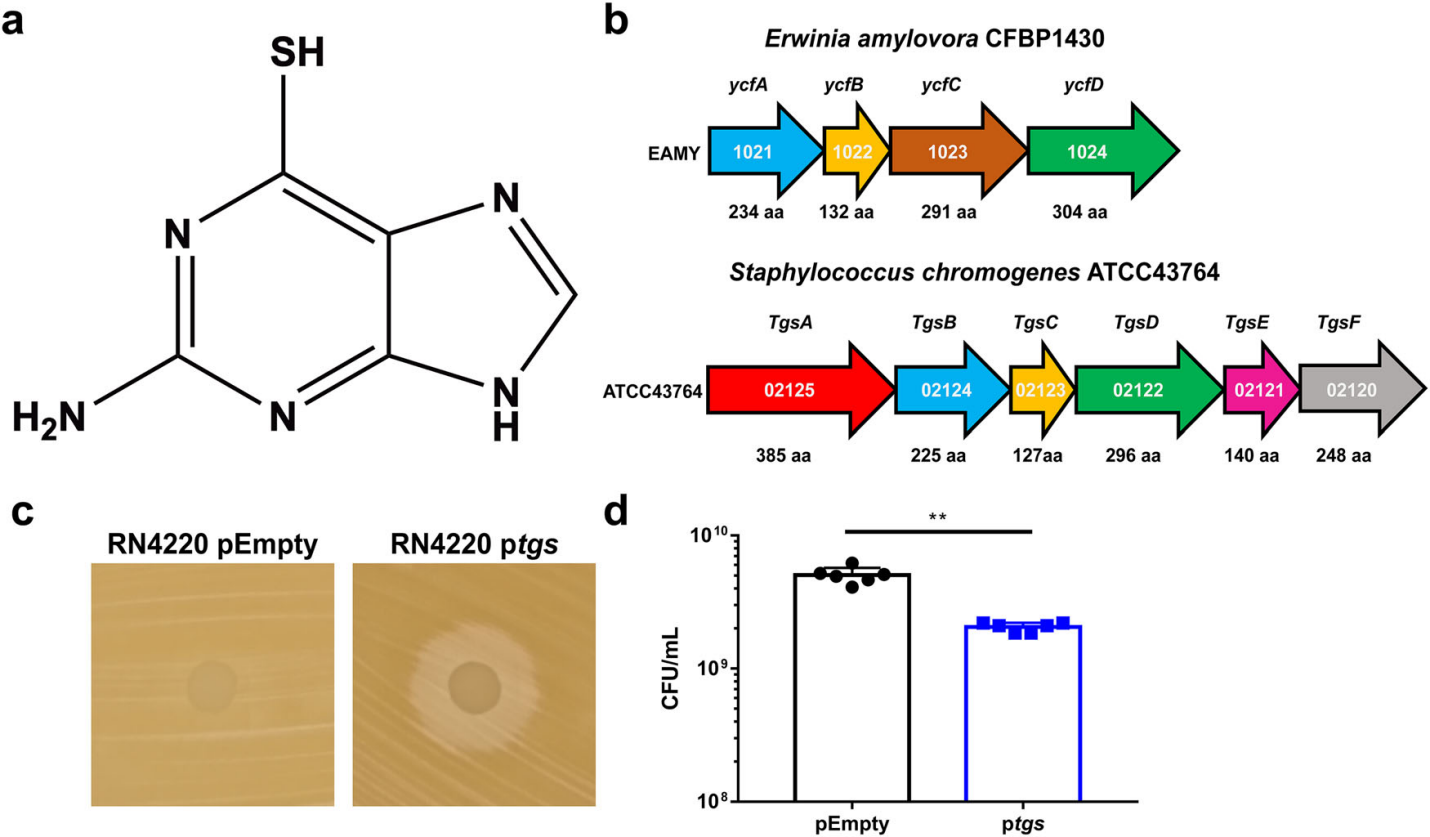
Identification of a 6-TG biosynthetic gene cluster in *S. chromogenes*. **a** Molecular structure of 6-thioguanine. **b** *E. amylovora ycf* operon and *S. chromogenes* 6-TG biosynthetic operon. **c** Simultaneous antagonism assay using RN4220 pEmpty and p*tgs* on TSA. **d** *S. aureus* was cultured in TSB containing supernatant from RN4220 pEmpty or p*tgs* and plated 24 h.p.i and CFU/mL were enumerated. Results are pooled from two independent experiments, each with *n* = 3 replicates per condition. Data are shown as mean ± SD (**p ≤ 0.01. Two-sided unpaired *t*-test).

To identify CoNS that antagonize the community-acquired MRSA strain *S. aureus* USA300-LAC (hereafter termed *S. aureus*), we screened a number of CoNS using the simultaneous antagonism assay^3^ (Supplementary Table 1 and Fig. 1a). Among the species and strains screened, we found that *S. chromogenes* strain ATCC43764, the type strain for the species, inhibited *S. aureus* growth on tryptic soy agar (TSA). We next demonstrated that the inhibitory molecule was secreted as spent culture supernatant (hereafter termed supernatant) of *S. chromogenes* also inhibited growth of *S. aureus* in liquid culture (Fig. 1b). The secreted molecule with inhibitory activity was found to be <3 kDa and resistant to heat and proteases (Supplementary Fig. 1A–D). For identification, the active molecule in *S. chromogenes* supernatant was enriched using sequential steps of centricon filtration, FPLC, and, finally, HPLC. Following HPLC, several contiguous fractions antagonized *S. aureus* growth equally (Supplementary Fig. 2A). These fractions were then analyzed by mass spectrometry in three independent experiments (Supplementary Table 2) to identify molecules present. We calculated a “score ratio” of these molecules to determine the relative abundance of these compounds across contiguous fractions within each experiment and found three molecules present in relatively equal abundance in biologically active fractions between experiments. Using these criteria, 6-TG (tioguanine; thioguanine), cinnamic acid, and indoleacrylic acid were the best candidate molecules (Supplementary Table 2). Indoleacrylic acid exerts anti-inflammatory activity and promotes intestinal barrier function^18^ and, based on this, we surmised it was unlikely to antagonize *S. aureus* growth. Interestingly, 6-TG and cinnamic acid have previously been shown to inhibit bacterial growth^19–21^. While cinnamic acid had no inhibitory effect on *S. aureus*, 6-TG impaired *S. aureus* growth on TSA (Supplementary Fig. 2B). Taken together these data suggest that the inhibitory molecule secreted by *S. chromogenes* is 6-TG (Fig. 2a).

### Identification of the 6-TG biosynthetic gene cluster in *S. chromogenes* ATCC43764

Next we sought to determine the genetic basis of 6-TG biosynthesis in *S. chromogenes* ATCC43764. To date, only *Erwinia amylovora*, a Gram-negative plant pathogen, has been shown to synthesize 6-TG, attributable to the four-gene *ycf* operon^22,23^. Hence, we sequenced and annotated the *S. chromogenes* ATCC43764 genome to mine for encoded proteins with predicted functional domains similar to YcfA and YcfB from *E. amylovora*, which are necessary and sufficient for 6-TG biosynthesis^23^. Using InterPro to predict functional protein domains, we identified Rossman-like alpha/beta/alpha folds and an adenine nucleotide hydrolase-like domain in YcfA and NADH pyrophosphatase and NUDIX hydrolase domains in YcfB. Interestingly, we found two gene products with identical functional domains within a predicted six-gene operon in the *S. chromogenes* ATCC43764 genome (Fig. 2b). Examination of the predicted products of each of the six genes within the operon (see Table 1) suggest roles in sulfur transfer, nucleotide metabolism, and secretion/efflux.

**Table 1:** Putative function of gene products in the *S. chromogenes* 6-TG biosynthetic cluster.

Protein	Predicted function	e.g., homolog; organism	Confidence (%)	Coverage (%)
TgsA	PLP-dependent cysteine desulfurase	SufS; *Bacillus subtilis*	100	96
TgsB	Nucleotide alpha hydrolase; sulfur transferase	TtuA; *Thermus thermophilus*	100	97
TgsC	NUDIX hydrolase family protein; DNA mismatch repair; pyrophosphatase	MutT; *Bacillus halodurans*	99.9	100
TgsD	Membrane protein; efflux	EmrE; *E. coli*	100	95
TgsE	NUDIX hydrolase family protein; DNA mismatch repair; 8-oxo-dGTP diphosphatase	MutT; *Mycobacterium smegmatis*	99.9	89
TgsF	Haloacid dehalogenase-like hydrolase; phosphatase	YfnB; *Bacillus subtilis*	100	92

Phyre^2^, the protein homology/analogy Recognition Engine v.2.0, web portal was used to search the protein structure databases for predicting protein structure and function.

To prove this operon in *S. chromogenes* was responsible for synthesizing 6-TG, we attempted to delete it from the *S. chromogenes* genome. Unfortunately, we were unable to transform the bacterium using existing plasmids and thus we were unable to genetically modify *S. chromogenes* using traditional molecular genetic methodologies. In lieu of this, we thus amplified and then cloned the genes from *S. chromogenes* into a plasmid and introduced this recombinant plasmid into *S. aureus* RN4220, creating a strain termed RN4220 p*tgs*. Remarkably, we showed that RN4220 p*tgs* inhibited *S. aureus* growth on TSA while RN4220 carrying an empty vector (termed RN4220 pEmpty) did not impair *S. aureus* growth (Fig. 2c). Moreover, supernatant from RN4220 p*tgs* inhibited *S. aureus* growth whereas supernatant from RN4220 pEmpty did not (Fig. 2d). Mass spectrometry was used to confirm that supernatants from RN4220 p*tgs*, but not RN4220 pEmpty, contained 6-TG (Supplementary Fig. 2C). Taken together, these data identify the genetic basis of 6-TG biosynthesis in *S. chromogenes* ATCC43764 and confirm that the expression of these genes by this species is detrimental to the growth of *S. aureus*. The six-gene operon was thus designated *tgsA-F*, for thioguanine synthesis.

### Identification of the *tgs* operon in other staphylococcal species

**Fig. 3:**
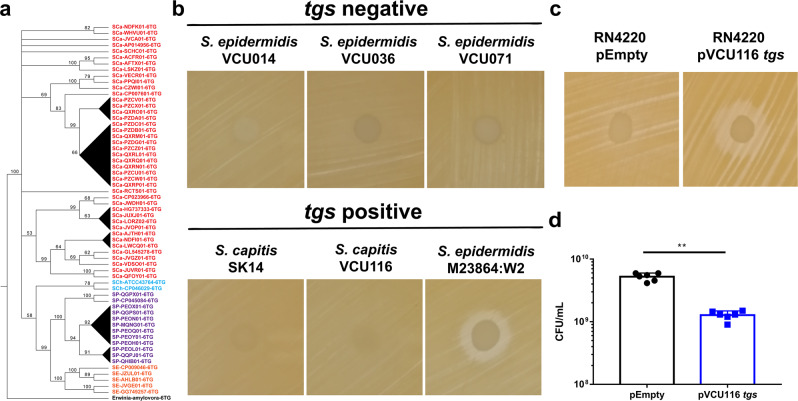
The *tgs* operon is present in other staphylococcal species and functional. **a** Phylogenetic analysis of the 6-TG operon using completed and draft *Staphylococcus* genomes. Black triangles represent a clade that was collapsed because their internal bootstrap values were <50%. SCa *S. capitis*, SCh *S. chromogenes*, SE *S. epidermidis*, SP *S. pseudintermedius*. **b** Simultaneous antagonism assay of *tgs*-negative and -positive staphylococcal strains on TSA. **c** Simultaneous antagonism assay using RN4220 pEmpty and pVCU116 *tgs* on TSA. **d** *S. aureus* was cultured in TSB containing supernatant from RN4220 pEmpty or pVCU116 *tgs* and plated 24 h.p.i and CFU/mL were enumerated. Results are pooled from two independent experiments, each with *n* = 3 replicates per condition. Data are shown as mean ± SD (**p ≤ 0.01. Two-sided unpaired *t*-test).

Next, we assessed the diversity of the *tgs* operon and its homologs in publicly available genomes of *Staphylococcus* species. We surveyed 1388 genomes across seven different species of *Staphylococcus* and discovered that the operon and its homologs can be found in genomes from *S. capitis*, *S. chromogenes*, *S. epidermidis*, and *S. pseudintermedius* (Table 2 and Supplementary Data 1). A phylogenetic analysis of these sequences showed that the *tgs* operon grouped into species-specific clades, suggesting that the operon was acquired by a common ancestor of each species and was then passed down via vertical inheritance (Fig. 3a). Interestingly, in *S. chromogenes* strain 1401, the operon is present on a plasmid, suggesting that it has the potential to be passed between different strains and species via horizontal gene transfer.

**Table 2:** The *tgs* operon among staphylococcal species.

Species	Total analyzed	Total *tgs*-positive	% total positive
*Staphylococcus capitis*	84	81	96.43
*Staphylococcus caprae*	10	0	0.00
*Staphylococcus chromogenes*	106	6	5.66
*Staphylococcus epidermidis*	804	6	0.75
*Staphylococcus hominis*	116	0	0.00
*Staphylococcus lugdunensis*	34	0	0.00
*Staphylococcus pseudintermedius*	234	65	27.75
Totals	1388	158	11.38

Distribution of isolates surveyed for the *tgs* operon and its homologs using tBLASTn.

To determine whether other CoNS that encode the *tgs* operon inhibit *S. aureus* growth in vitro, we tested both *tgs-*negative and -positive strains that were identified in our bioinformatic analyses using the simultaneous antagonism assay. As expected, the *tgs*-positive *S. epidermidis* M23864:W2 strain inhibited growth of *S. aureus* while the *tgs-*negative strains did not. However, both *tgs*-positive *S. capitis* strains did not inhibit *S. aureus* growth in our assay conditions (Fig. 3b). This result led us to hypothesize that expression of the *tgs* operon in these strains was repressed. Thus, to test this hypothesis, we amplified and cloned the *tgs* operon from one of the *S. capitis* strains (VCU116) into a plasmid, and introduced this recombinant plasmid into *S. aureus* RN4220, creating a strain termed RN4220 pVCU116 *tgs*. Of note, RN4220 pVCU116 *tgs* inhibited *S. aureus* growth while RN4220 pEmpty did not (Fig. 3c, d). Mass spectrometry confirmed that both RN4220 pVCU116 *tgs* and *S. epidermidis* M23864:W2 produce 6-TG. Moreover, supernatants from both *tgs*-positive *S. capitis* strains had detectable levels of 6-TG, although ~20-fold less than that of RN4220 pVCU116 *tgs* or the other *tgs*-positive species that showed growth-inhibitory effects on *S. aureus* (Supplementary Fig. 3A). Therefore, it is likely that the *S. capitis* strains did not inhibit *S. aureus* growth due to low amounts of secreted 6-TG. Next, we assessed whether expression of the *tgs* operon from both *S. chromogenes* and *S. capitis* is detrimental to RN4220, a *S. aureus* cloning strain. Both RN4220 p*tgs* and RN4220 pVCU116 *tgs* show a growth defect compared to RN4220 pEmpty, revealing that 6-TG production is detrimental to the growth of the producing *S. aureus* strain RN4220 (Supplementary Fig. 3B).

### Expression of *tgs* genes ameliorates *S. aureus* virulence in vivo

**Fig. 4:**
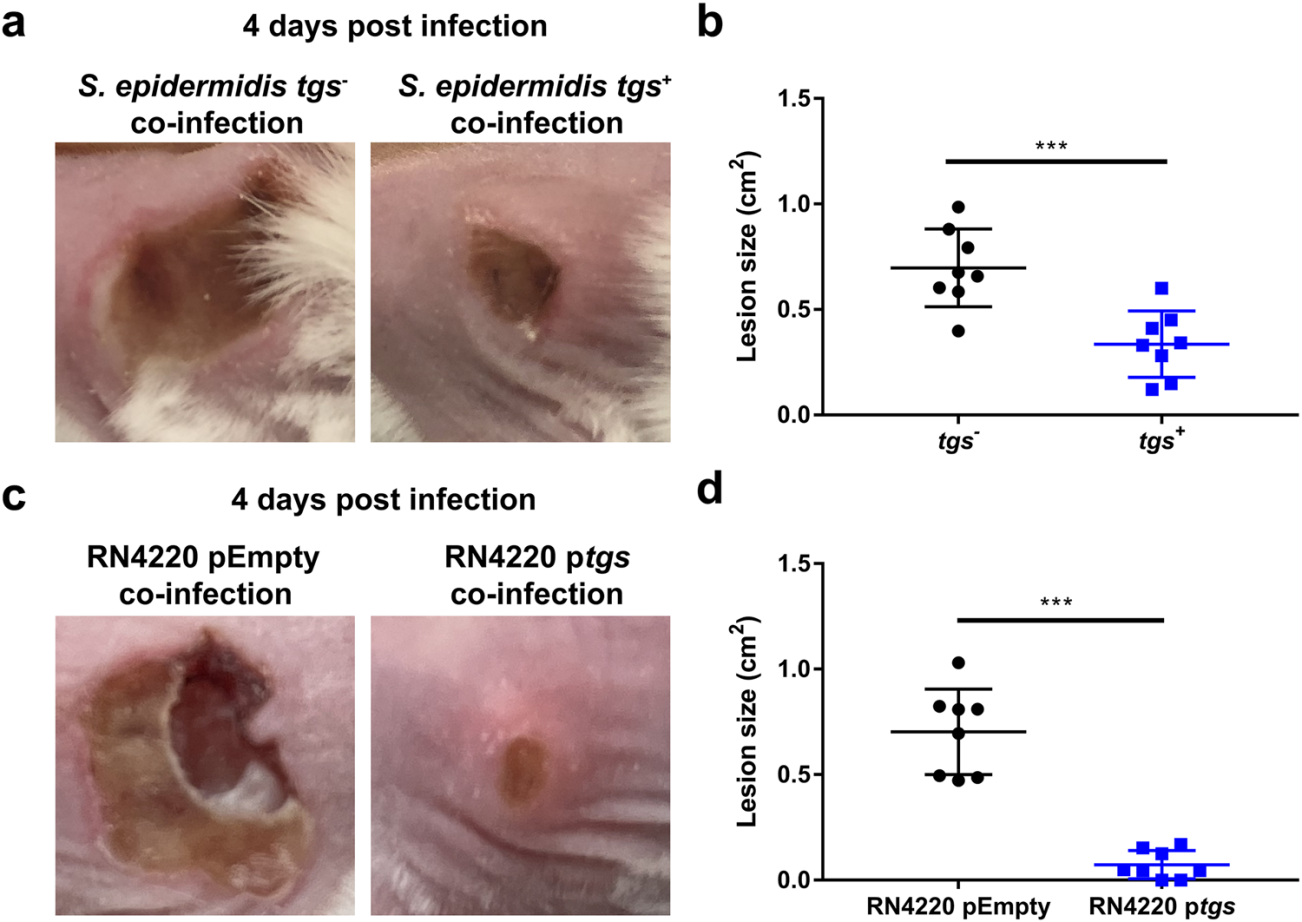
*tgs* expressing staphylococci reduce skin damage caused by *S. aureus* during co-infection. **a** Representative images showing necrotic skin lesions 4 days post infection with *S. aureus* + *tgs-*negative *S. epidermidis* str. VCU036 or *S. aureus* + *tgs*-positive *S. epidermidis* str. M23864:W2 in a 1:1 ratio. **b** Quantitation of necrotic skin lesion size 4 days post infection with *S. aureus* + *tgs*-negative *S. epidermidis* str. VCU036 or *S. aureus* + *tgs*-positive *S. epidermidis* str. M23864:W2 in a 1:1 ratio. Results are from one representative experiment enumerating 8 lesions per group (*n* = 4 animals per group). Data are shown as mean ± SD (***p ≤ 0.001. Two-sided unpaired *t*-test). **c** Representative images showing necrotic skin lesions 4 days post infection with *S. aureus* + RN4220 pEmpty or *S. aureus* + RN4220 p*tgs* in a 1:1 ratio. **d** Quantitation of necrotic skin lesion size 4 days post infection with *S. aureus* + RN4220 pEmpty or *S. aureus* + RN4220 p*tgs* in a 1:1 ratio. Results are from one experiment enumerating 8 lesions per group (*n* = 4 animals per group). Data are shown as mean ± SD (***p ≤ 0.001. Two-sided unpaired *t*-test).

Our results demonstrated that staphylococci that express the *tgs* operon inhibit *S. aureus* growth in vitro. To determine whether these strains can offer protection to the host and antagonize *S. aureus* in vivo, we performed co-infection experiments using *S. aureus* USA300 and *tgs*-negative or *-*positive *S. epidermidis* strains in a subcutaneous infection model, as these species are known to co-colonize on human skin^11,24^. We mixed *S. aureus* with either a *tgs*-negative or *-*positive *S. epidermidis* strain in a 1:1 ratio and measured the size of lesions over time. Importantly, we observed that co-infections with a *tgs-*positive *S. epidermidis* strain reduced skin damage caused by *S. aureus* compared to co-infections with a *tgs*-negative *S. epidermidis* strain (Fig. 4a, b). Admittedly, these experiments using different *S. epidermidis* strains were correlative. However, to demonstrate a causative effect of *tgs* genes, we repeated these co-infection experiments by combining *S. aureus* USA300 (produces necrotic lesions) with *S. aureus* RN4220 (does not produce necrotic lesions) carrying either pEmpty or p*tgs*. Remarkably, we observed that co-infections with RN4220 p*tgs* resulted in significantly smaller lesions compared to co-infections with RN4220 pEmpty (Fig. 4c, d). These data show that staphylococci that express the *tgs* operon can protect the host against necrotic skin infections caused by *S. aureus*.

### 6-TG inhibits tissue necrosis during subcutaneous *S. aureus* infection

**Fig. 5:**
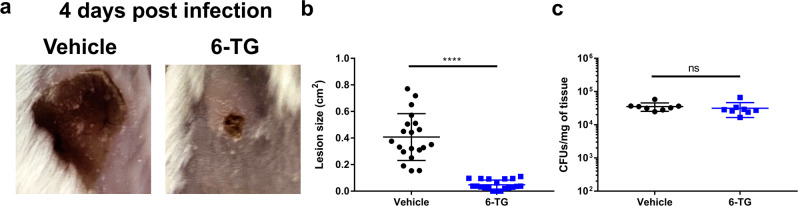
Prophylactic treatment with 6-TG reduces necrotic lesion size in subcutaneous infection. **a** Representative images showing necrotic skin lesions 4 days post infection with *S. aureus* ± prophylactic 6-TG treatment. **b** Quantitation of necrotic skin lesion size 4 days post infection with *S. aureus* ± prophylactic 6-TG (20 µg) treatment. Results are pooled from two independent experiments with 8–12 lesions per group (*n* = 4 and 6 animals, respectively). Data are shown as mean ± SD (****p ≤ 0.0001. Two-sided unpaired *t*-test). **c** Skin tissue was excised from mice infected with *S. aureus* 4 days post infection ± prophylactic 6-TG treatment (20 µg) and plated onto TSA to enumerate CFUs. Results are from one experiment with 8–12 lesions per group (*n* = 4 and 6 animals, respectively). Data are shown as mean ± SD (two-sided unpaired *t*-test).

Although co-infections with *tgs*-positive strains can protect the host against skin damage caused by *S. aureus*, it is unlikely that these strains can be administered as a probiotic to combat *S. aureus* infections as these bacteria may also cause infections on their own. Therefore, we sought to assess the therapeutic effect of 6-TG on formation of necrotic skin lesions in a subcutaneous *S. aureus* infection. To do this, we prophylactically administered 6-TG subcutaneously and measured the size of lesions throughout infection. Strikingly, we observed that the necrotic lesions of treated mice were significantly smaller than the lesions on mice injected with vehicle control (Fig. 5a, b). Moreover, we observed a reduction in bacterial load when we excised the lesions of mice treated with 6-TG relative to the mice given the vehicle control (Supplementary Fig. 4). However, in additional and in subsequent experiments, and irrespective of lesion size, if we excised similar areas of skin from both the vehicle and 6-TG-treated mice we found that, despite significant differences in lesion size between treated vs. untreated groups, similar bacterial burdens were present at 4 days post infection (Fig. 5c). The discrepancy in the measured bacterial load using these two methods suggest that quantification of CFUs is more accurate when a large section of the skin is excised, irrespective of the lesion size. Taken together, these data demonstrate that prophylactic treatment with 6-TG significantly reduces necrotic lesion formation caused by *S. aureus*.

### 6-TG blocks toxin production in *S. aureus* in a purine-dependent and -independent manner

6-TG is a guanine analog that is known to inhibit purine biosynthesis^20,25^. In support of the notion that 6-TG inhibits *S. aureus* growth by interfering with purine biosynthesis, we show that a *S. aureus purK* mutant, with a disrupted purine biosynthesis pathway^26^, was largely resistant to 6-TG-mediated growth inhibition on TSA (Supplementary Fig. 5A). Furthermore, 6-TG is more potent against *S. aureus* grown on purine-deplete RPMI than on TSA (Supplementary Fig. 5A, B). Indeed, *S. aureus* growth was partially rescued by addition of guanine in RPMI (Supplementary Fig. 5B). Interestingly, 6-TG is also active against strains representing other *S. aureus* clonal lineages, although to varying degrees (Supplementary Fig. 6). Taken together, these data indicate that 6-TG primarily impairs *S. aureus* growth by inhibiting purine biosynthesis.

**Fig. 6:**
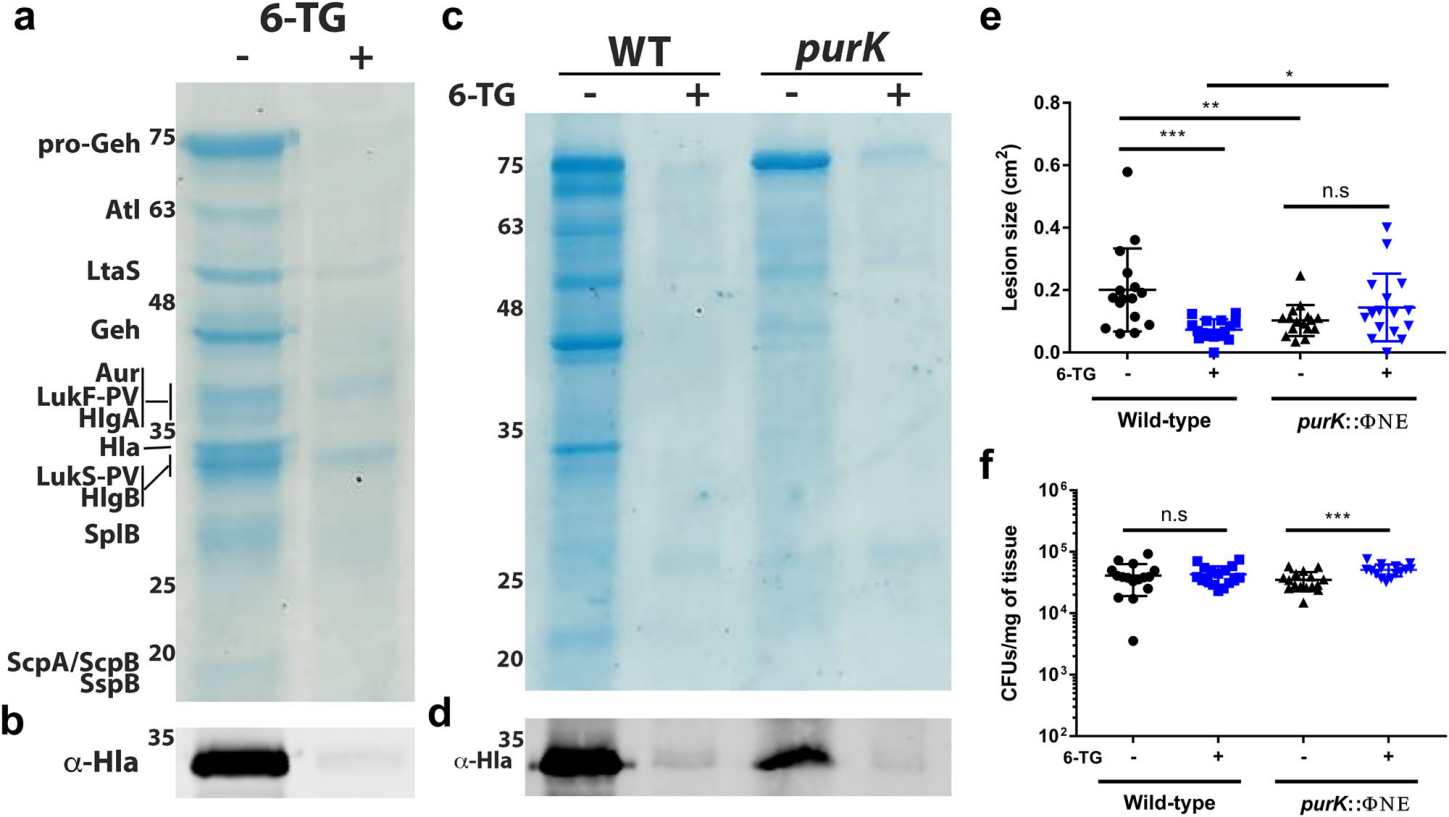
6-TG reduces *S. aureus* toxin production in a purine-dependent and -independent manner. **a** *S. aureus* was grown in the presence of 6-TG (10 µg/mL) or a vehicle control to an OD_600_ of 7.0 and secreted proteins in the supernatant were TCA precipitated and separated by SDS-PAGE. This experiment was repeated three times with similar results. **b** Protein samples in **a** were immunoblotted and probed with anti-alpha toxin antibody in a Western blot. Protein identification was by MALDI TOF MS. Numbers indicate molecular mass markers. This experiment was repeated three times with similar results. **c** *S. aureus* and *S. aureus purK*::ΦΝΣ was grown in TSB with 6-TG (10 µg/mL) or a vehicle control to an OD_600_ of 7.0 and secreted proteins in the supernatant were TCA precipitated and separated by SDS-PAGE. This experiment was repeated twice with similar results. **d** Protein samples in **c** were immunoblotted and probed with anti-alpha toxin antibody in a Western blot. This experiment was repeated twice with similar results. **e** Quantitation of necrotic skin lesion size 4 days post infection with *S. aureus* or *S. aureus purK*::ΦΝΣ ± prophylactic 6-TG (20 µg) treatment. Results are from one representative experiment with 16 lesions enumerated per group (*n* = 8 animals). Data are shown as mean ± SD (***p ≤ 0.001, **p ≤ 0.01, *p ≤ 0.05. Two-sided unpaired *t*-test). **f** Skin tissue was excised from mice infected with *S. aureus* or *S. aureus purK*::ΦΝΣ 4 days post infection ± prophylactic 6-TG (20 µg) treatment and plated onto TSA to enumerate CFUs. Results are from one representative experiment with 16 lesions enumerated per group (*n* = 8 animals). Data are shown as mean ± SD (***p ≤ 0.001. Two-sided unpaired *t*-test). For **a–d**, numbers to the left of gels/blots are molecular mass markers (in kDa).

Since 6-TG abrogated necrosis in the *S. aureus* subcutaneous infection model, we questioned whether the compound could curtail production of *S. aureus* secreted toxins, which are required for tissue necrosis in this model^27^. Interestingly, several studies demonstrate a link between purine biosynthesis and *S. aureus* virulence^20,26,28–31^. To determine whether 6-TG, a purine biosynthesis inhibitor, reduces toxin production, we cultured *S. aureus* in tryptic soy broth (TSB) with 6-TG or a vehicle control to stationary phase (OD_600_ = 7.0), collected supernatants and analyzed the secreted protein profile using SDS-PAGE. We observed that supernatants from bacteria grown with 6-TG had less overall protein, including several toxins, compared to vehicle treated cultures (Fig. 6a). Next, we chose Hla as a representative *S. aureus* toxin and evaluated the effects of 6-TG on its levels in *S. aureus* cultures. Importantly, this toxin is known to be important for *S. aureus* pathogenesis in skin infections^27,32,33^. Here, we used an anti-Hla antibody to confirm that Hla levels were dramatically reduced when *S. aureus* was exposed to 6-TG (Fig. 6b), and demonstrated that this reduction in Hla was dose-dependent and correlated with its effect on growth (Supplementary Fig. 7A–C).

To determine whether toxin production was abrogated by 6-TG due to its effect on purine biosynthesis, we performed SDS-PAGE on culture supernatants from *S. aureus* and *purK*::ΦΝΣ grown in TSB with 6-TG or a vehicle control. Interestingly, we observed that *purK*::ΦΝΣ secretes less overall protein compared to its wild-type counterpart in the absence of 6-TG, confirming that purine biosynthesis is linked to toxin production in *S. aureus*. However, *purK*::ΦΝΣ secretes less overall protein when cultured with 6-TG despite already being deficient in purine biosynthesis (Fig. 6c). A western blot analysis demonstrated that these findings also extend to Hla protein levels in cell culture supernatants (Fig. 6d). We confirmed that the effects of 6-TG on *purK*::ΦΝΣ were not due to an effect on growth, as the compound does not affect the mutant’s growth kinetics (Supplementary Fig. 8). Overall, these results suggest that 6-TG affects toxin production in both a purine-dependent and -independent manner.

Next, we evaluated the importance of *S. aureus* purine biosynthesis for necrotic lesion formation in a subcutaneous infection. Here, we prophylactically treated mice with 6-TG or a vehicle control before subcutaneously infecting them with *S. aureus* or *purK*::ΦΝΣ. As expected, *purK*::ΦΝΣ caused smaller necrotic lesions compared to wild-type *S. aureus* in mice given a vehicle control (Fig. 6e). However, the lesion size caused by this mutant was unaffected by 6-TG, suggesting that a very-low amount of Hla is required for lesion formation and a significant increase in Hla levels is required to increase lesion size (i.e., the relationship between Hla levels and lesion size is not completely linear). Interestingly, *purK*::ΦΝΣ treated with 6-TG had a slight increase in bacterial burden relative to the control without treatment (Fig. 6f). These data reveal the importance of purine biosynthesis for the regulation of toxin production in *S. aureus* in a subcutaneous infection model.

### 6-TG abolishes transcription of ribosomal genes and genes of the *pur* and *agr* operons

**Fig. 7:**
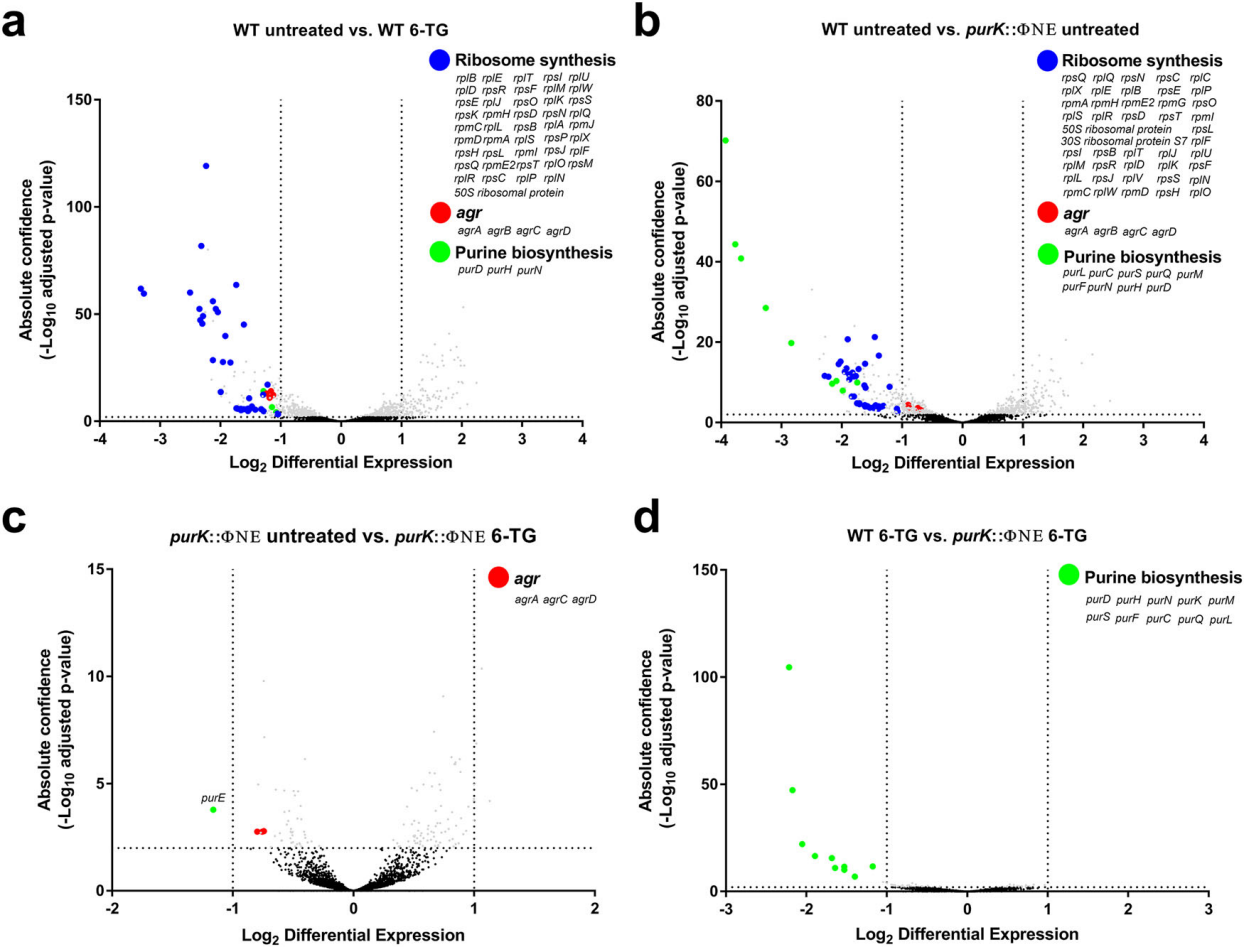
6-TG abrogates toxin production by reducing transcription from the agr locus and from genes encoding for ribosomal proteins. **a** RNA-seq analysis of wild-type *S. aureus* cultures treated with a vehicle control or 6-TG (10 µg/mL) in TSB. Data points above the dashed lines along the *x*-axis represent *p* ≤ 0.01. Data points outside of the dashed lines along the *y*-axis represent fold changes of ≥2.0. Results are from four independent experiments. **b** RNA-seq analysis of *S. aureus purK*::ΦΝΣ cultures treated with a vehicle control compared to wild-type *S. aureus* cultures treated with a vehicle control in TSB. Results are from four independent experiments. **c** RNA-seq analysis of *S. aureus purK*::ΦΝΣ cultures treated with a vehicle control or 6-TG (10 µg/mL) in TSB. Results are from four independent experiments. **d** RNA-seq analysis of wild-type *S. aureus* cultures and *S. aureus purK*::ΦΝΣ cultures treated with 6-TG (10 µg/mL) in TSB. Results are from four independent experiments. Statistical significance was determined using a negative binomial distribution. Detailed RNA-seq data can be found in Supplementary Data 2.

Next, we wanted to determine the exact changes that 6-TG exerts on *S. aureus* and to understand how it affects toxin production in both a purine-dependent and -independent manner. To accomplish this, we cultured both wild-type *S. aureus* and its *purK*::ΦΝΣ derivative to exponential phase (OD_600_ = 1.0) and exposed them to 6-TG for 1 h before collecting the cells and extracting the RNA to perform RNA sequencing (RNA-seq). Some of the most upregulated genes upon exposure to 6-TG were CodY-regulated genes, such as branched chain amino acid synthesis genes. We attribute this to the effects 6-TG has on repressing guanine levels and, thus, guanosine triphosphate (GTP), which is an effector molecule of CodY DNA binding and thus its transcriptional repression^34^. However, to understand how 6-TG inhibits toxin production in *S. aureus*, we focused our analyses on transcripts that were repressed by 6-TG. As expected, transcripts from the *pur* operon were significantly downregulated in wild-type cells treated with 6-TG, owing to the drug’s effect on purine biosynthesis. Surprisingly, transcripts from the *agr* operon and over 40 genes that encode for ribosomal proteins were also significantly reduced by 6-TG in wild-type cells (Fig. 7a). These results suggest that 6-TG abrogates toxin production in *S. aureus* in two ways; it downregulates transcription from the global virulence regulator *agr* and it globally reduces protein synthesis by preventing ribosomal proteins from being synthesized, which consequently impedes toxin production. Indeed, 1-h treatment with 6-TG already reduces the expression of *hla* by 1.65-fold (40%) (Supplementary Data 2). To determine which effects are purine-dependent or -independent, we compared transcript levels between WT and *purK*::ΦΝΣ in cultures treated and untreated with 6-TG (Fig. 7b). Strikingly, there was a strong overlap in genes that were affected by the *purK* mutation in *S. aureus* (Fig. 7b), versus WT treated with 6-TG (Fig. 7a). Indeed, there was a significant reduction in transcripts from the *pur* operon because *purK*::ΦΝΣ is inherently deficient in purine synthesis. However, the number of transcripts from genes that encode for many ribosomal proteins was also reduced compared to wild-type cells, suggesting a link between purine biosynthesis and ribosome synthesis. Interestingly, transcripts from the *agr* operon are reduced to a lesser extent in the purine biosynthesis mutant compared with WT that has been treated with 6-TG (Fig. 7b, c.f. a), suggesting that the effects 6-TG has on *agr* are not fully purine-dependent. In support of this, transcripts from *agr* are reduced further when the *purK*::ΦΝΣ culture is treated with 6-TG relative to the untreated *purK*::ΦΝΣ culture (Fig. 7c). No significant differences in the number of transcripts were found when comparing the transcriptome of the *purK*::ΦΝΣ mutant and wild-type cells when both cultures were treated with 6-TG (Fig. 7d). Altogether, these data show that 6-TG drastically reduces ribosome synthesis in a purine-dependent manner and blocks *agr* activation in both a purine-dependent and -independent manner. As a result, production of proteinaceous toxins will be significantly diminished as translation is blocked by 6-TG.

## Discussion

*S. aureus* is a major human pathogen that causes a spectrum of mild to severe diseases, including self-limiting skin infections to sepsis^1^. The emergence of MRSA has sparked interest in discovering new drugs to combat drug-resistant *S. aureus* infections. Attention has been focused on CoNS for drug discovery as these species compete against *S. aureus* in their respective ecological niches. Indeed, CoNS including *S. epidermidis, S. hominis, S. lugdunensis*, and *S. capitis*, have been reported to secrete effector molecules to inhibit *S. aureus* colonization and reduce its virulence^9–11,35^. These studies illustrate the importance of CoNS as a potential source of compounds that can be used to treat *S. aureus* infections.

In this work, we found a strain of *S. chromogenes* that blocks purine biosynthesis to inhibit *S. aureus* growth. Importantly, de novo purine biosynthesis has recently been shown to regulate the virulence potential of *S. aureus*^26,30,31^. It is interesting that the *tgs* operon was found in *S. chromogenes* because this is a species that co-colonizes with *S. aureus* in cattle^36–38^. Since these two species occupy the same ecological niche, it is conceivable that they are in constant competition for space and nutrients. Indeed, it has been suggested that the presence of *S. chromogenes* offers protection against *S. aureus* infections in bovines^36^. The conservation of the *tgs* operon in *S. chromogenes* and other CoNS suggests that the genes have moved during competition in niches they co-occupy. For example, the CoNS that harbor the *tgs* operon colonize skin and mucosal surfaces of various mammals including humans, similar to *S. aureus* and are known to cause opportunistic infections^24,39,40^. The *tgs*-positive *S. chromogenes* and *S. epidermidis* strains that inhibit *S. aureus* growth produce at least tenfold more 6-TG into the culture supernatant than the two *tgs*-positive *S. capitis* strains. This difference would appear to account for the observed difference in the growth-inhibitory ability between the species, since the *S. capitis tgs* genes produce enough 6-TG to inhibit *S. aureus* growth when they are expressed from a multi-copy plasmid. Our ongoing studies seek to define the regulatory mechanism and signal(s) controlling expression of the operon in *S. capitis*.

Here, we showed that NUDIX domain-containing proteins are responsible for 6-TG biosynthesis. NUDIX domain proteins are widespread among microbes and have been implicated in bacterial pathogenesis^41^. Therefore, it is likely that microbes besides *Erwinia* and staphylococcal species also synthesize 6-TG. At this time, the enzymatic reactions required to synthesize 6-TG are unknown without confirmation of biochemical activity of the Tgs enzymes. Our bioinformatic analyses (Table 1) allow us to speculate that GTP can serve as an initial substrate. A combination of TgsC, TgsE, and/or TgsF may cleave the phosphates off GTP to generate guanosine. TgsA is predicted to release a sulfur group from a donor molecule (e.g., cysteine), which then likely serves as a substrate for TgsB, which we predict transfers it guanine. TgsD is a predicted efflux membrane protein and presumably exports 6-TG out of the cell, and likely also therefore provides self-resistance to the molecule. Interestingly, our data demonstrate that, despite possessing the *tgsD* gene, growth of recombinant *S. aureus* carrying the *tgs* operon is still slowed in comparison to strains carrying vector alone (Supplementary Fig. 3).

In this work, we show that bacteria expressing the *tgs* operon can reduce lesion formation in a subcutaneous *S. aureus* infection in a 6-TG-dependent manner during a co-infection. These data along with other studies demonstrate that CoNS on the skin can provide protection against infections caused by the more pathogenic *S. aureus* on the skin^9,11,14^. To demonstrate the therapeutic potential of 6-TG, we showed that the pure compound can be used to prophylactically treat *S. aureus* skin infections. Our data suggest that 6-TG acts therapeutically by reducing the overall amount of toxin that *S. aureus* may secrete during infection. We show that the molecular basis for the therapeutic effect of 6-TG is likely due to its ability to abrogate purine biosynthesis, which subsequently reduces ribosome synthesis and toxin production. Moreover, 6-TG downregulates transcription of the global virulence regulator *agr*, further reducing toxin production. By inhibiting de novo purine biosynthesis, we show that 6-TG can act as an anti-virulence compound in a subcutaneous *S. aureus* skin infection model as purine synthesis and toxin production are linked. These data reveal purine biosynthesis as an attractive drug target for treatment of MRSA infections, as targeting this pathway significantly affects toxin production, which is important for many types of *S. aureus* infections at different sites of the body^42^. Thus, it will be interesting to determine whether 6-TG can also be used to reduce disease severity and/or bacterial burden in other models of infection in future studies. Indeed, Lan et al. have shown that 6-TG can reduce *S. aureus* burden in a systemic infection in mice^20^. Moreover, it will be important to test other nucleobase analogs against *S. aureus* to potentially expand our repertoire of antimicrobials and/or anti-virulence compounds to treat MRSA infections.

Like other thiopurines, 6-TG is a pro-drug that enters the purine salvage pathway and is converted into its active form, 6-thioguanosine monophosphate, by the enzyme hypoxanthine-guanine phosphoribosyl transferase (HPRT) in humans^43^. Once converted into an active metabolite, 6-TG is thought to block purine synthesis and be incorporated into DNA, causing cytotoxic effects in eukaryotic cells^25,44^. Whether 6-TG affects bacteria by exerting mutagenic effects is currently unknown. However, Sun et al. showed that 6-TG interacts with *Mycoplasma pneumoniae* HPRT to block purine salvage^21^. This suggests that 6-TG may also antagonize *S. aureus* through the purine salvage pathway by being activated by HPRT. We are currently investigating the molecular targets of 6-TG and to identify determinants that make *S. aureus* susceptible to 6-TG.

It is well known that certain antibiotics can select for *agr* mutations and *S. aureus* strains harboring these mutations become more resistant to these stresses and cause chronic infections^45^. However, we currently have no evidence to suggest that 6-TG selects for the emergence of these mutants or that *agr*-negative strains are resistant to 6-TG. In this study, we observed that the *agr*-negative strain RN4220 is susceptible to the growth-inhibitory effects of 6-TG. Future work will seek to identify mechanisms of resistance against 6-TG in *S. aureus* and how evolving resistance against this drug affects *S. aureus* physiology and metabolism.

It is becoming increasingly clear that many CoNS, including the species identified in this study that contain the *tgs* operon, co-colonize with *S. aureus* and competition between these species for the same niches can provide protection for the host. This work reveals a previously unknown mechanism by which the relatively understudied CoNS can inhibit growth and virulence of more pathogenic bacteria like *S. aureus*. This, in turn, should spark interest in the discovery of additional genes synthesizing other nucleotide analogs that may be used by bacteria in inter-species bacterial warfare.

## Methods

### Bacterial strains and plasmids

Bacterial strains and plasmids are listed in Supplementary Table 1. Unless otherwise noted, strains were cultured at 37 °C with 200 RPM shaking. *E. coli* was grown in Luria broth (LB) or on LB agar containing 100-μg/mL ampicillin to maintain plasmids when necessary. *S. aureus* USA300 and RN4220 were grown in TSB or on TSB agar plates (TSA). Staphylococcal plasmids were maintained with growth in 10-μg/mL chloramphenicol when necessary.

### Bacterial supernatant growth inhibition assays

Overnight cultures of *S. chromogenes* and *S. aureus* were grown in TSB for ~24 h. Cultures were pelleted at 21,000 g for 5 min and supernatants were filter-sterilized using a 0.22μm syringe filter. The OD_600_ of *S. aureus* cultures was normalized to 1.0 and 10 μl (corresponding to 0.01 ODU, ~5 × 10^6^ CFUs) were grown in a 1.0-mL mixture of TSB and either *S. chromogenes* or *S*. *aureus* spent culture supernatants (as indicated in figure or legend) in 13-mL tubes at 37 °C with 200 RPM shaking for 24 h. Bacterial cultures were serially diluted and plated onto TSA to enumerate CFUs at 24 h post inoculation.

### *S. chromogenes* supernatant treatments

For the use of Amicon^®^ Ultra centrifugal filter units, filter-sterilized supernatants were centrifuged at 3620 *g* for 20 min to separate the supernatant into filtrate and concentrate. TSB was added to increase the concentrate volume to the original volume of the supernatant. For heat treatment, supernatants were filter-sterilized and incubated in a 95 °C heat block for 1 h. For proteinase K treatment, 10 μL of a 20-mg/mL proteinase K solution was added to 1 mL of filter-sterilized supernatant and incubated in a 37 °C heat block for 1 h. Proteinase K was inactivated at 95 °C for 30 min. For trypsin treatment, 20 μL of a 20-mg/mL trypsin solution was added to 1 mL of filter-sterilized supernatant and incubated in a 37 °C heat block for 1 h. Trypsin was inactivated at 95 °C for 30 min.

### Fast protein liquid chromatography

Twenty milliliter of *S. chromogenes* <3-kDa supernatant was loaded on a Sigma-Aldrich HiTrap^®^ SP Fast Flow column on an Amersham Pharmacia Biotech AKTA FPLC system. Samples were eluted in a 0–50% gradient of solvent B (50-mM Tris pH 8.0) over solvent A (20-mM Tris pH 7.5). Five milliliter fractions were collected at a flow rate of 2.0 mL/min.

### High performance liquid chromatography

Ten milliliter of the active fraction from FPLC was concentrated using a SpeedVac vacuum concentrator and re-suspended in 300-μl ddH_2_O. Two hundred microliter of the suspension was injected into a preparative Waters Bondapak C18 column on a Waters Breeze HPLC. Samples were eluted in a 0–100% gradient of solvent B (methanol or acetonitrile) over solvent A (water or water with 0.1% trifluoroacetic acid). Two milliliter fractions were collected at a flow rate of 4.0 mL/min.

### Liquid chromatography–mass spectrometry for identification of compounds

A 10-μl injection of each fraction was eluted from a Thermo Scientific Hypersil Gold C18 column at 40 °C on a Thermo Scientific Ultimate 3000 UHPLC, with a binary solvent system containing water with 0.1% (v/v) formic acid (solvent A) and acetonitrile containing 0.1% (v/v) formic acid (solvent B) at a 0.3-mL/min flow rate. The gradient initiated with a 1.0-min hold at 5% B. From 1.0 to 7.0 min, the gradient increased linearly to 98% B followed by a hold for 3 min. The gradient then decreased linearly to 5% B from 10–10.5 min and held at 5% B for 4.5 min. The Thermo Scientific Q Exactive mass spectrometer was operated using the following settings: Ion source, HESI II; spray voltage, 3.5 kV; sheath gas flow, 15; auxiliary gas, 5; spare gas, 2; capillary temperature, 320 °C; S-lens RF level, 50. A scan range of 100–1200 was used to identify ions in both positive and negative modes. An untargeted MS_2_ experiment was performed using an isolation window of 1.0 *m*/*z* and a normalized collision energy of 30 (arbitrary units). Untargeted data processing was done using Thermo Compound Discoverer 2.1.

### Identification of candidate inhibitory compounds

Each compound identified using mass spectrometry in every fraction was given a score based on its abundance in their respective fractions; a higher score signifies higher abundance. Compounds were not considered if they were not identified in all three experiments. Next, we divided the score from the fraction with the highest score (i.e., most concentrated) by the one with the lowest score (i.e., least concentrated) for each compound. The formula for each compound was calculated as follows: score ratio = (compound A highest score)/(compound A lowest score). A score ratio of a molecule approaching a value of 1 signifies an equal distribution of that compound between active fractions. Here, we chose to consider hits with a score ratio of 10 or less in all three experiments as potential candidates for the inhibitory molecule. We chose this cut-off for the score ratio under the assumption that the inhibitory compound was evenly distributed throughout the active fractions since inhibitory activity was equal between these fractions.

### Whole-genome sequencing of genomic DNA

*S. chromogenes* genomic DNA was isolated by phenol–chloroform extraction. The extracted genomic DNA for H278Tor was tagmented using the Nextera XT tagmentation kit (Illumina) according to manufacturer protocol. The tagmented products were sequenced on an Illumina MiniSeq with paired-end sequencing (2 × 150 bp) at the University of Toronto. The extracted genomic DNA for H278Pit, H279, JB98, and JB383 was tagmented using the Nextera tagmentation kit (Illumina) using a modified protocol^46^. The tagmented products were sequenced on an Illumina NextSeq 550 with paired-end sequencing (2 × 150 bp) at the Microbial Genome Sequencing Center in Pittsburgh, PA. The “Pit” and “Tor” designations for H278 refer to the sequencing location for the isolates to differentiate between the two during downstream analyses. DNA sequence reads were trimmed using Trimmomatic version 0.36^47^ with the following parameters: ILLUMINACLIP, NexteraPE:2:30:10:8:true; LEADING, 20; TRAILING, 20; SLIDINGWINDOW, 4:20; MINLEN, 36. The reads were assembled using the SPAdes version 3.13 assembler^48^ in careful mode. Contigs for both the whole genome and the plasmid were annotated using Prokka version 1.12^49^.

### Bioinformatic analyses of the 6-TG operon in *Staphylococcus* species

Draft and completed genomes of seven different *Staphylococcus* species (*capitis*, *caprae*, *chromogenes*, *epidermidis*, *hominis*, *lugdunensis*, and *pseudintermedius*) were downloaded from NCBI. The 6-TG operon and its homologs were identified using tBLASTn^50^ using translation table 11, with the *S. chromogenes* 6-TG operon used as the query sequence. Genomes that contained the operon are listed in Supplementary Data 1. The 6-TG operon and its homologs were extracted, and their nucleotide sequences were aligned in Geneious Prime 2020.1.2 (https://www.geneious.com) using the Geneious aligner and the default settings. The alignment was used to create a phylogeny with RAxML using the rapid bootstrapping and search for best maximum likelihood tree algorithm with 1000 bootstrap iterations^51^. The RAxML tree was condensed with MEGA7^52^ using a bootstrap support cut-off of 50%. Three isolates possessed split 6-TG operons and were excluded from the phylogenetic analysis to minimize potential problems due to operon assembly.

### Cloning of the 6-TG biosynthetic cluster from *S. chromogenes* and *S. capitis* into *S. aureus*

Genomic DNA was isolated from *S. chromogenes* and *S. capitis* by phenol–chloroform extraction. Primers containing a *KpnI* or *SacI* restriction site were used to amplify the putative 6-TG biosynthetic cluster (TTTTTTGGTACCCCGAACTTTTATTCGAAAAT and TTTTTTGAGCTCCTAGTTTTTAATTGTAAAAC) from *S. chromogenes* ATCC43764 and (TTTTTTGGTACCTGTTTCTTTTACTTGTCTGC and TTTGAGCTCGGTTATTTTCAGTACACTCC) from *S. capitis* VCU116 (Supplementary Table 3). The amplified product was ligated into pALC2073 that was similarly digested and transformed into *E. coli*. Plasmids isolated from *E. coli* were introduced into *S. aureus* RN4220.

### Simultaneous antagonism and disc diffusion assays

The OD_600_ of bacteria was normalized to 1.0. Sterile cotton swabs were used to spread plate a suspension of the culture onto solid media. For simultaneous antagonism assays, 10 μL of overnight bacterial cultures were inoculated onto the bacterial lawn. For modified simultaneous antagonism assays, a well in solid media was generated and 100 μL of a suspension was inoculated into the well. For disc diffusion assays, sterile paper discs were placed on top of the bacteria and 10 μL of 10-mg/mL 6-TG dissolved in 0.1-M NaOH was dropped onto the disc. For cinnamic acid, 10 μL of 10-mg/mL cinnamic acid was dissolved in 20% ethanol and dropped onto the disc. Plates were incubated at 37 °C overnight.

### Assessing the effect of the expression of the *tgs* operon on *S. aureus* growth kinetics

Single colonies of RN4220 pEmpty, RN4220 p*tgs* and RN4220 pVCU116 *tgs* were inoculated into 5-mL TSB supplemented with chloramphenicol and grown overnight. Thirty milliliter of TSB supplemented with chloramphenicol were inoculated with bacteria from the overnight cultures such that the starting OD_600_ was 0.01. The OD_600_ of the cultures was measured every hour for the first 8 h. The OD_600_ was then measured at the 12th and 24th hour post inoculation.

### Co-infections between *S. aureus* and *tgs*-positive and *tgs*-negative strains

Eight-to-ten-week-old female Balb/c mice were shaved 1 day before the day of infection. Overnight cultures (grown in TSB) of *S. aureus* USA300, *S. epidermidis* str. VCU036, *S. epidermidis* str. M23864: W2, RN4220 pEmpty and RN4220 p*tgs* were subcultured to OD_600_ of 0.05 in TSB and grown to OD_600_ of 2.0–2.2. Bacterial cells were pelleted and washed in phosphate-buffered saline (PBS) twice. Bacterial cells were then normalized to the appropriate OD_600_ and 25 μL of the *S. aureus* bacterial suspension (~5.0 × 10^7^ CFUs) was mixed with 25 μL of the corresponding bacterial suspension (~5.0 × 10^7^ CFUs for each strain) and subcutaneously injected into the animals. Infected mice were monitored daily for 4 days and sacrificed at 96 h post infection. Lesion sizes were analyzed in ImageJ.

### 6-TG prophylactic treatment of *S. aureus* subcutaneous skin infections

Eight-to-ten-week-old female Balb/c mice were shaved and subcutaneously injected with 25 μL of 800 μg/mL 6-TG (equivalent to 20 μg 6-TG or 1 mg/kg) or a vehicle control 1 day before infection. This dosage was chosen based on the therapeutic concentration of 6-TG used in both mice and in humans for inflammatory bowel disease^53,54^. Overnight cultures (grown in TSB) of *S. aureus* USA300 were subcultured to OD_600_ of 0.05 in TSB and grown to OD_600_ of 2.0–2.2. Bacterial cells were pelleted and washed in PBS twice. Bacterial cells were then normalized to OD_600_ 3.7 and 25 μL of the bacterial suspension (~5.0 × 10^7^ CFUs) were mixed with 25 μL of 800 μg/mL 6-TG and subcutaneously injected into the animals. Infected mice were monitored daily for 4 days and sacrificed at 96 h post infection. Lesions were excised in PBS with 0.1% (v/v) triton X-100, homogenized in a bullet blender tissue homogenizer (2 × 5 min at speed 6) and serially diluted before being plated onto TSA to enumerate CFUs. Lesion sizes were analyzed in ImageJ.

### Assessing the effect of 6-TG on *S. aureus* protein secretion by SDS-PAGE

Single colonies of *S. aureus* grown on TSA were inoculated into TSB with 10-μg/mL 6-TG or a vehicle control and grown to an OD_600_ of ~7.0–8.0. Proteins in the supernatant were harvested through trichloroacetic acid (TCA) precipitation. Briefly, supernatant derived from 6.0 OD_600_ units of bacterial culture were mixed with an equal volume of ice-cold 20% (w/v) TCA for 3 h, washed twice with ice-cold 70% ethanol and air dried. The proteins were dissolved in 1x Laemmli SDS-PAGE reducing buffer and boiled for 10 min. Proteins were separated on a 12% SDS-PAGE gels and stained with InstantBlue^TM^ Ultrafast Protein Stain overnight for visualization of protein bands.

### Assessing the effect of 6-TG on *S. aureus* alpha toxin by western blot

Western blot analysis on alpha toxin was performed by transferring the SDS-PAGE gel to a nitrocellulose membrane. The membrane was then blocked with PBS supplemented with 0.1% (v/v) Tween 20 and 5% (w/v) milk. After blocking, the membrane was incubated with rabbit anti-Hla (1:500) polyclonal antibody (Sigma-Aldrich) for 2 h and washed with PBS-Tween 20 (3x). Donkey anti-rabbit IRDye 800 (1:20,000) (Rockland) was used as a secondary antibody for 45 min and the membrane was washed with PBS-Tween 20 (3x) prior to imaging. Membranes were scanned using an Odyssey Clx imaging system (Li-Cor Biosciences). Supernatants from both wild-type *S. aureus* and a Δ*sbi*Δ*spa* derivative were used, and the same results were obtained with either strain.

### Assessing the effect of 6-TG on *S. aureus* supernatant hemolysis

Single colonies of *S. aureus* grown on TSA were inoculated into TSB. The overnight culture was subcultured into fresh TSB supplemented with a vehicle control or different concentrations of 6-TG. After 24 h of growth, the culture supernatants were centrifuged and filter-sterilized. Ten microliter of the cell-free supernatant was inoculated onto TSA supplemented with 5% (v/v) human red blood cells. Images were taken after 24 h and the area of the zone of hemolysis was measured using ImageJ.

### Assessing the effect of 6-TG on *S. aureus* growth kinetics

Single colonies of *S. aureus* and *S. aureus purK*::ΦΝΣ were inoculated into 5-mL TSB and grown overnight. Thirty milliliter of TSB with 10 μg/mL of 6-TG or a vehicle control were inoculated with bacteria from *S. aureus* or *S. aureus purK*::ΦΝΣ overnight cultures such that the starting OD_600_ was 0.01. The OD_600_ of the cultures was measured every hour for 24 h.

### Preparation of cells for RNA extraction

Single colonies of *S. aureus* and *S. aureus purK*::ΦΝΣ were inoculated into 5-mL TSB and grown overnight. Five milliliter of TSB were inoculated with bacteria from *S. aureus* or *S. aureus purK*::ΦΝΣ overnight cultures such that the starting OD_600_ was 0.01. Cultures were grown until OD_600_ = 1.0 (3–3.5 h) and 10-μg/mL 6-TG or a vehicle control was added to the culture. Cultures were grown for another hour. An equivalent of OD_600_ = 6.0 of cells was collected and RNAprotect Bacteria Reagent (Qiagen) was mixed with the cells as per manufacturer’s instructions. Cell pellets were frozen overnight at −80 °C.

### RNA extraction and RNA-seq

To extract RNA from frozen *S. aureus* and *S. aureus purK*::ΦΝΣ, cell pellets were washed with TE buffer (pH 8.0) and re-suspended in a Tris-HCl mixture with 1-mg/mL lysostaphin (pH 7.5) to lyse for 1 h at 37 °C. RNA was extracted using an Aurum^TM^ Total RNA Mini Kit (BioRAD #7326820) as per the manufacturer’s instructions. TURBO^TM^ DNase (ThermoFisher Scientific) was used to cleave contaminating DNA, and RNA was further purified by phenol/chloroform extraction. Concentration and purity of RNA were determined using a Nanodrop. RNA samples were processed by the Microbial Genome Sequencing Center in Pittsburgh, PA. Ribosomal RNA was depleted (Qiagen FastSelect5S/16S/23S), and cDNA libraries were generated (Qiagen Total Stranded RNA). cDNA libraries were sequenced using NextSeq 550 using a 75 × 8 × 8bp setup.

### Bioinformatic analyses of RNA-seq data

Sequencing reads were mapped to the reference *S. aureus* USA300 FPR3757 genome in Geneious Prime 2020.1.2 (https://www.geneious.com). Expression analysis and comparisons were performed using DESeq2^55^.

### Graphical and statistical analyses

Statistical analyses were performed using GraphPad Prism software version 7. Statistical significance was calculated using ANOVA and Student’s *t* tests with SD as appropriate.

### Ethics approval

All experiments involving animals were reviewed and approved by the Animal Use Subcommittee of the University of Western Ontario and were performed according to the Canadian Council on Animal Care guidelines.

### Reporting summary

Further information on research design is available in the Nature Research Reporting Summary linked to this article.

## Supplementary information

Supplementary Information

Description of Additional Supplementary Files

Supplementary Data 1

Supplementary Data 2

Reporting Summary

## Data Availability

The raw sequence reads for the *S. chromogenes* ATCC43764 (H278Tor, H278Pit, H279), JP98 and JP383 isolates sequenced in this study can be found in the NCBI Sequence Read Archive under BioProject ID: PRJNA630769. RNA-seq data can be found in the NCBI Sequence Read Archive under BioProject ID: PRJNA695221. The authors declare that the data supporting the findings of this study are available within the paper and its supporting Supplementary information files. Source data are provided with this paper.

## Source data

Source data
