# Supplementary material for: Coagulase-negative staphylococci release a purine analog that inhibits Staphylococcus aureus virulence
Source: Nat Commun. 2021 Mar 25;12:1887. doi: 10.1038/s41467-021-22175-3 (PMC7994395; doi:10.1038/s41467-021-22175-3)
Supplement: Supplementary file 1 — Supplementary Information [file 41467_2021_22175_MOESM1_ESM.pdf]

Supplementary Materials for

Coagulase-negative staphylococci release a purine analog that inhibits *Staphylococcus aureus* virulence

Denny Chin[1], Mariya I. Goncheva[1], Ronald S. Flannagan[1], Shayna R. Deecker[2], Veronica Guariglia-Oropeza[1], Alexander W. Ensminger[2] and David E. Heinrichs[1]*

Correspondence to: deh@uwo.ca

This PDF file includes:

Supplementary Figures 1 to 8.

Supplementary Tables 1 to 3

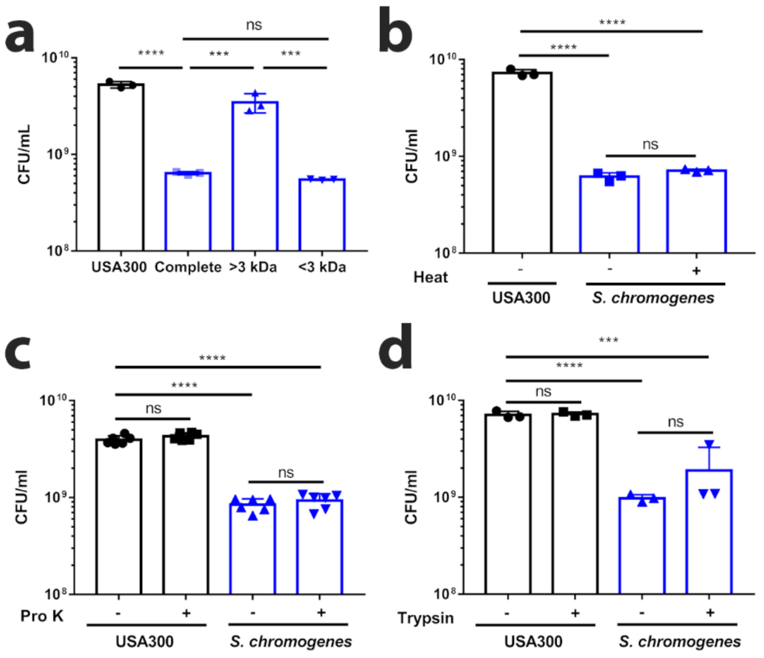

Supplementary Fig 1: The inhibitory molecule secreted by *S. chromogenes* is < 3 kDa and resistant to high temperatures and proteases

a) *S. aureus* was cultured in TSB containing fractionated (>3kDa or <3kDa) spent culture supernatant from *S. chromogenes* (1:1 v/v ratio) or a 1:1 v/v ratio of *S. aureus* spent culture supernatant (as indicated). Following 24 hr incubation at 37°C, bacteria were plated and CFU/mL were enumerated. Data are shown as mean ± SD (n = 3 biological replicates from one experiment, ***$p \leq 0.001$, ****$p \leq 0.0001$. One-way ANOVA with Tukey's multiple comparisons). **b, c, d)** *S. aureus* was cultured in TSB containing 1:1 v/v ratio of *S. aureus* spent culture supernatant (as indicated, or 1:1 v/v ratio of spent culture supernatant from *S. chromogenes* that was pre-treated with heat (**b**), proteinase K (**c**) or trypsin (**d**). Following 24 hr incubation at 37°C, bacteria were plated and CFU/mL were enumerated. Data are shown as mean ± SD (n = 3 biological replicates from one experiment **(a)**, **(b)**, **(d)** and n = 6 biological replicates from 2 independent experiments for **(c)**, *** $p \leq 0.001$, ****$p \leq 0.0001$. One-way ANOVA with Tukey's multiple comparisons).

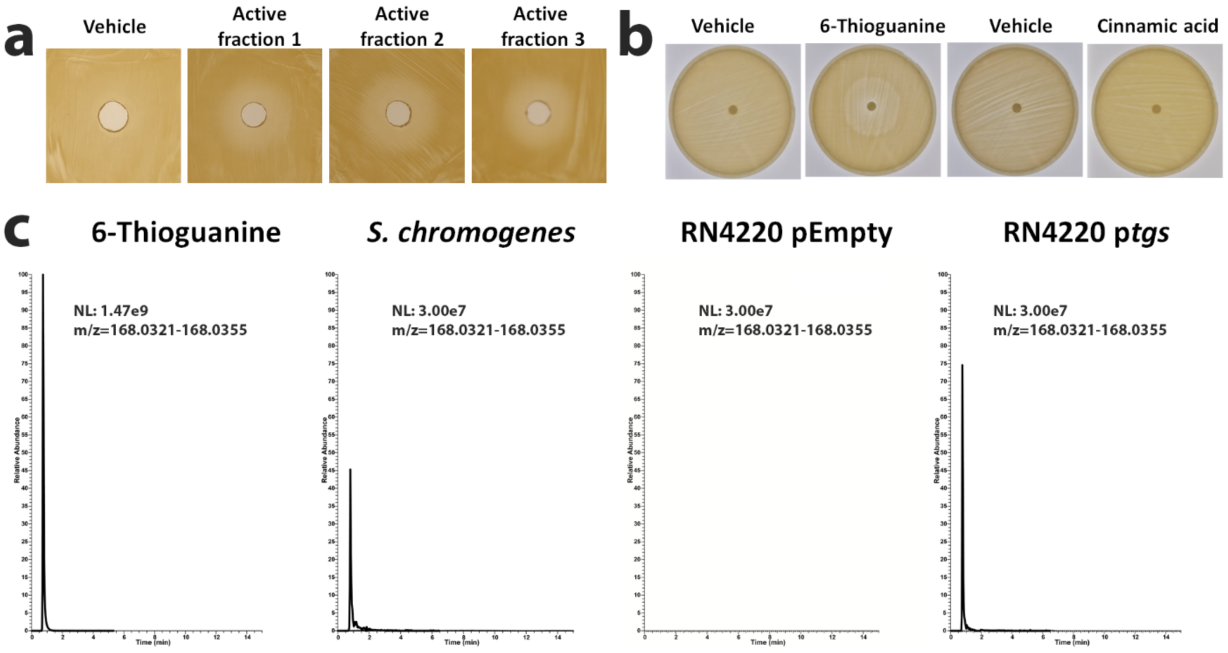

Supplementary Fig 2: The inhibitory molecule secreted by *S. chromogenes* is 6-thioguanine
a) Fractions obtained from HPLC were concentrated and inoculated into a well in TSA onto which *S. aureus* had been plated to form a lawn following 24 hr incubation at 37°C. **b)** 6-TG and cinnamic acid (10 µL of 10 mg/mL solution for each) were spotted onto paper discs overlaid on TSA onto which *S. aureus* had been plated to form a lawn following 24 hr incubation at 37°C. Zones of inhibition were compared to their respective vehicle controls. **c)** Targeted mass spectrometry to identify 6-TG in the samples (L to R): pure commercial 6-TG, and spent culture supernatants of *S. chromogenes* ATCC43764, RN4220 pEmpty, and RN4220 p*tgs*.

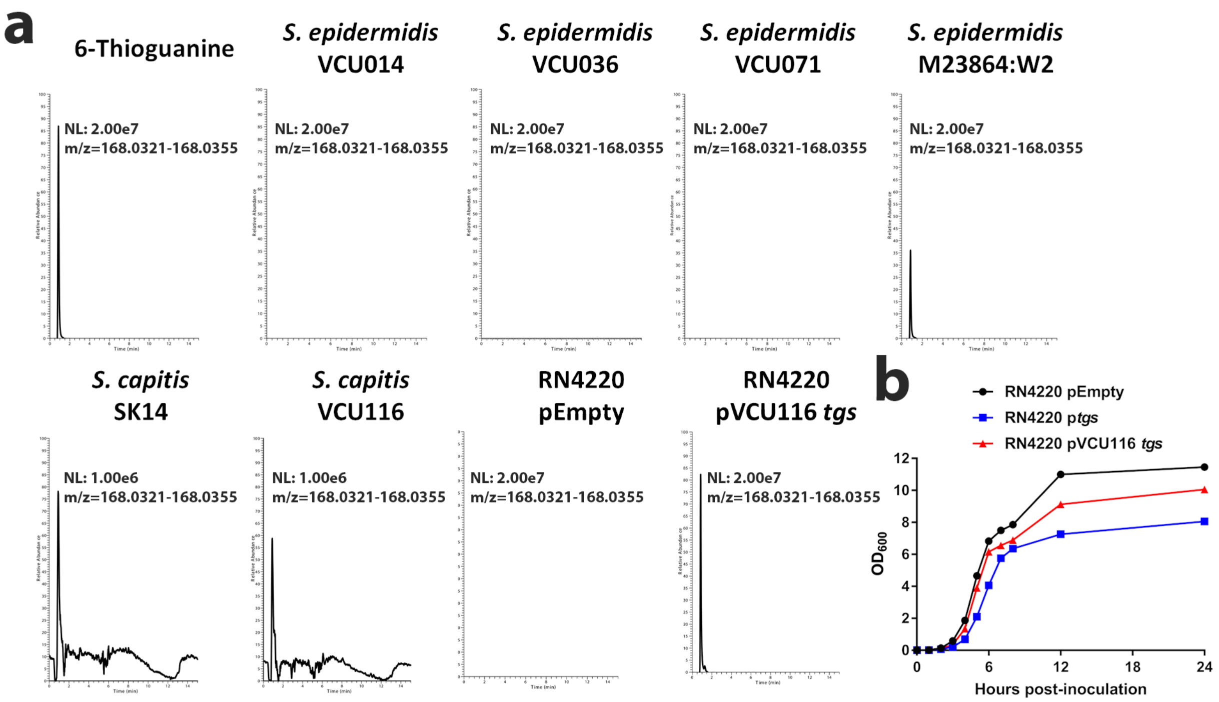

Supplementary Fig 3: Other CoNS that harbour the *tgs* operon secrete 6-TG

a) Targeted mass spectrometry was performed to identify 6-TG in spent culture supernatants from the indicated strains grown 24 hr in TSB. **b)** RN4220 pEmpty, RN4220 *ptgs* and RN4220 pVCU116 *tgs* cultures were grown at 37°C in TSB supplemented with chloramphenicol and OD_{600} was measured every hour for 24 hr. Results are pooled from two independent experiments with three biological replicates in total. Data are shown as mean ± SD.

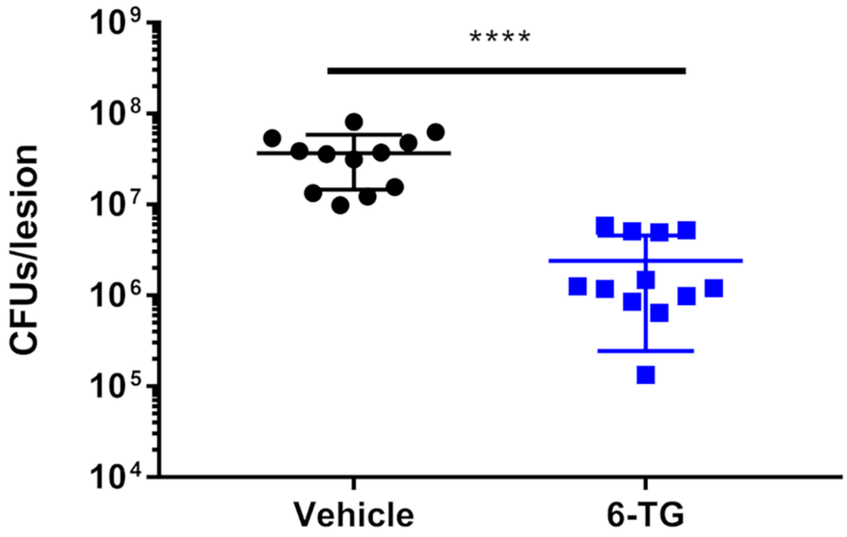

Supplementary Fig 4: Bacterial burden directly beneath necrotic lesions
Lesions were excised from mice infected with *S. aureus* 4 days post infection ± prophylactic 6-TG treatment and plated onto TSA to enumerate CFUs. Data are shown as mean ± SD (****p ≤ 0.0001. Two-sided unpaired t-test).

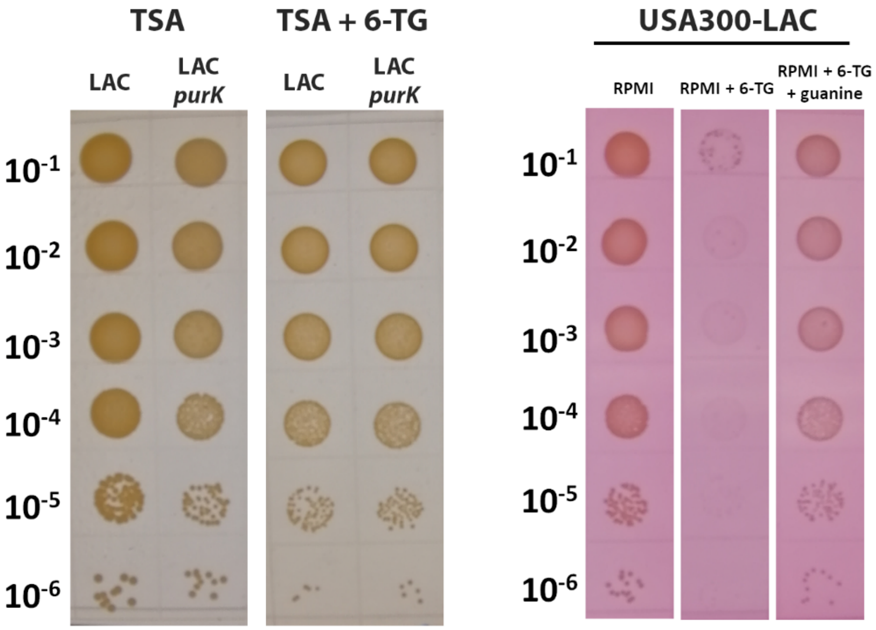

Supplementary Fig 5: 6-TG inhibits growth by blocking *de novo* purine biosynthesis
Bacterial strains (as indicated) were grown overnight at 37°C in TSB, the OD_{600} was normalized to 1.0, and then serially diluted and plated (10 µL drops) onto media as indicated. TSA supplemented with 10 µg/mL 6-TG (as indicated) or vehicle. Wildtype = *S. aureus* USA300 LAC.

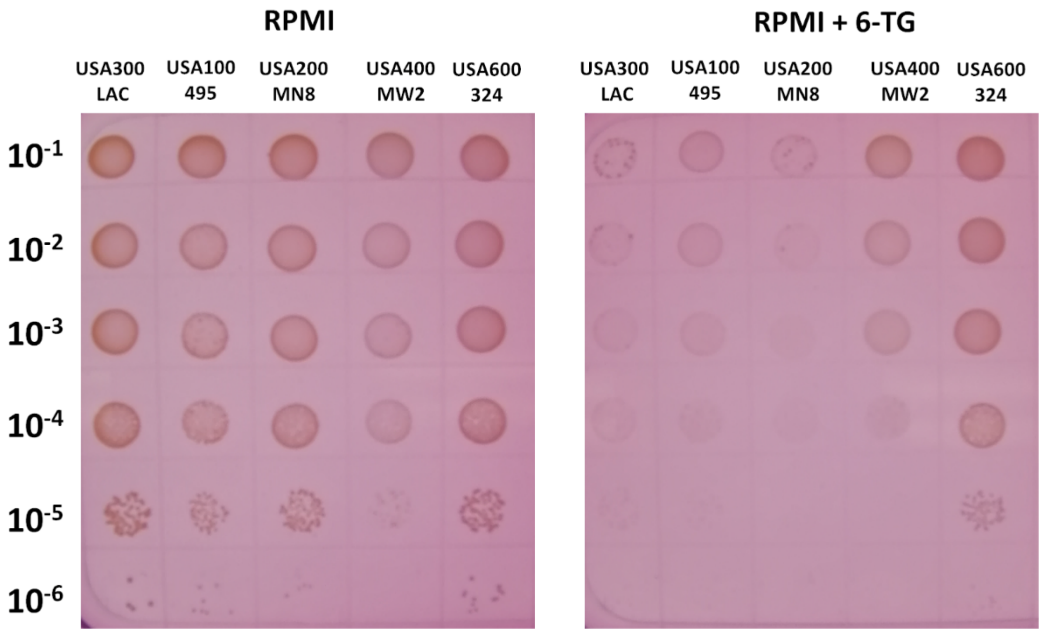

Supplementary Fig 6: Effect of 6-TG on growth of strains representing different clonal lineages of *S. aureus*

Bacterial strains (as indicated) were grown overnight at 37°C in TSB, the OD_{600} was normalized to 1.0, and then serially diluted and plated (10 µL drops) onto solid RPMI medium, with or without supplementation with 10 µg/mL 6-TG (as indicated).

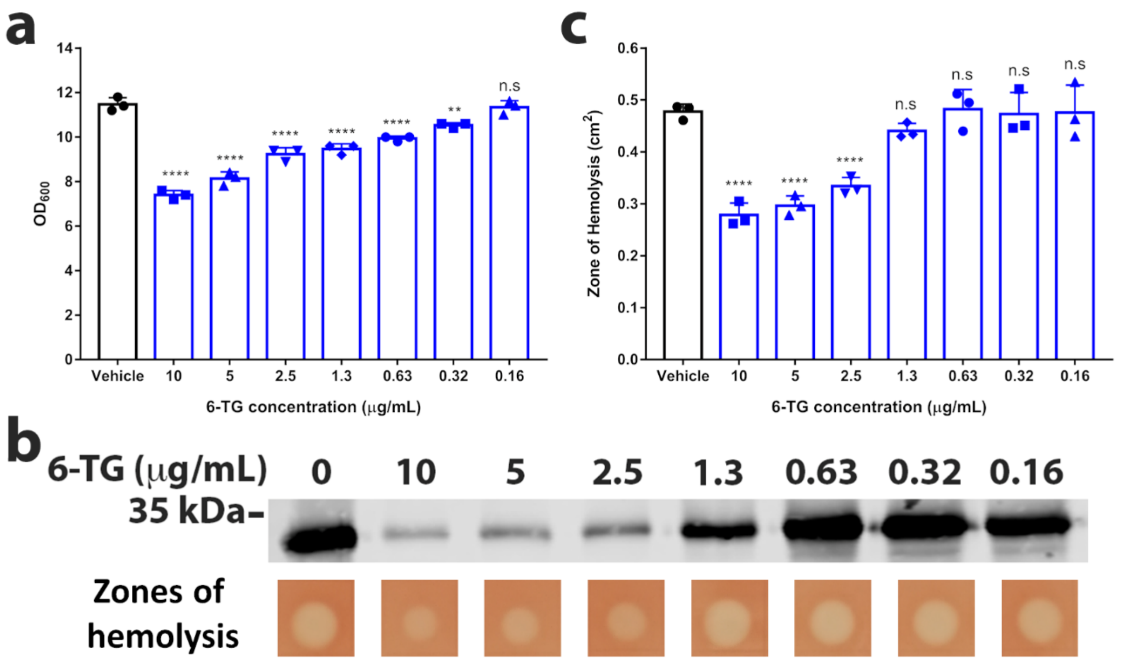

Supplementary Fig 7: 6-TG represses *S. aureus* growth and toxin production in a dose-dependent manner

a) *S. aureus* was grown 24 hr at 37°C in TSB containing varying concentrations of 6-TG, as indicated, and OD_{600} was measured. Data are shown as mean ± SD (n = 3 biological replicates from one representative experiment, ** $p \leq 0.01$, ****$p \leq 0.0001$. One-way ANOVA with Dunnett's multiple comparisons test). **b)** Cell-free supernatants from (A) were used to assess i) relative Hla production by Western blotting of TCA precipitated supernatant (top row) and ii) relative hemolytic activity of supernatant spotted onto TSA 5% human blood agar plates. Images were taken 24 hours after spotting. This experiment was repeated twice with similar results. **c)** Quantification of zones of hemolysis in **b)**. Data are shown as mean ± SD (n = 3 biological replicates from one representative experiment, ****$p \leq 0.0001$. One-way ANOVA with Dunnett's multiple comparisons test).

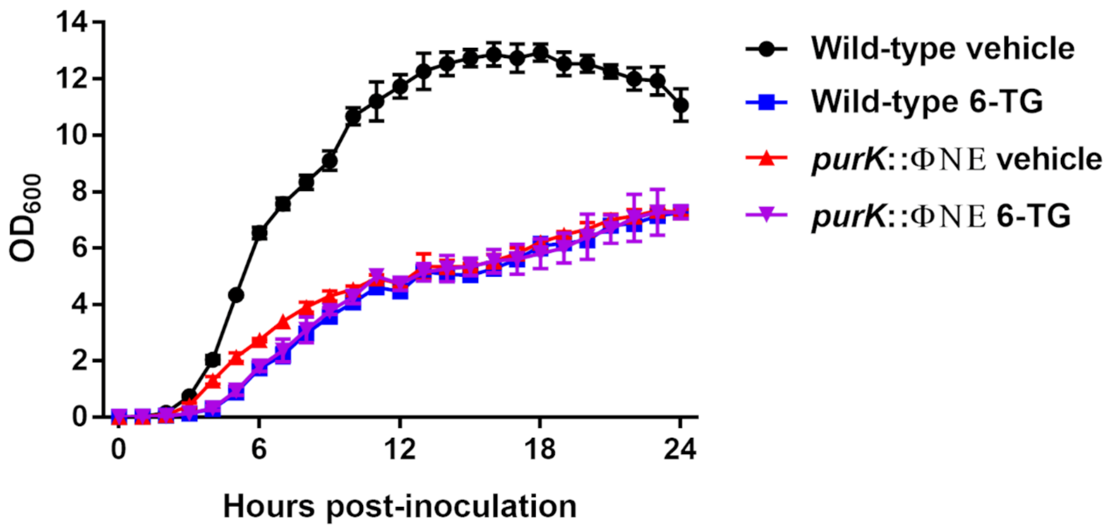

Supplementary Fig 8: *S. aureus purK*::ΦΝΣ growth is unaffected by 6-TG

S. aureus or *S. aureus purK*::ΦΝΣ were grown in TSB with 6-TG (10 µg/mL) or a vehicle control and the OD_{600} was measured every hour for 24 hours. Results are pooled from three biological replicates. Data are shown as mean ± SD.

Supplementary Table 1 – Bacterial strains used in this study.

Strain	Description	Source
S. chromogenes ATCC43764	*Staphylococcus chromogenes*	ATCC
S. aureus USA300	USA300 LAC, cured of resistance plasmids	Lab stock
S. aureus Δsbispa	USA300 LAC with a clean deletion in *sbi* and *spa*	1
S. aureus RN4220	r_{K}- m_{K+}; capable of accepting foreign DNA	Lab stock
S. aureus purK::ΦNΣ	USA300 with a transposon insertion in the *purK* gene	1
RN4220 pEmpty	RN4220 carrying pALC2073	This study
RN4220 p*tgs*	RN4220 carrying the 6TG biosynthetic operon from *S. chromogenes* in pALC2073	This study
RN4220 pVCU116 *tgs*	RN4220 carrying the 6TG biosynthetic operon from *S. capitis* str. VCU116 in pALC2073	This study
S. cohnii ATCC29973		ATCC
S. warneri ATCC27836		ATCC
S. warneri ATCC27837		ATCC
S. capitis ATCC35661		ATCC
S. sciuri ATCC29062		ATCC
S. arlettae		J.Scott Weese
S. epidermidis LK819		M. Valvano
S. epidermidis E66B-1		M. Valvano
S. epidermidis E55B-1		M. Valvano
S. epidermidis RMS846-1		M. Valvano
S. simulans ATCC27851		ATCC
S. xylosus ATCC35663		ATCC
S. hominis ATCC27846		ATCC
S. auricularis ATCC33753		ATCC
S. saprophyticus ATCC15305		ATCC
S. saprophyticus ATCC35552		ATCC
S. lugdunensis HKU09-01		2
S. lugdunensis N920143		3
S. capitis SK14	*tgs* positive	BEI resources
S. capitis VCU116	*tgs* positive	BEI resources
S. epidermidis M23864:W2	*tgs* positive	BEI resources
S. epidermidis VCU014	*tgs* negative	BEI resources
S. epidermidis VCU036	*tgs* negative	BEI resources
S. epidermidis VCU071	*tgs* negative	BEI resources
USA100 495		J. McCormick
USA200 MN8		ATCC
USA400 MW2		B. Kreiswirth
USA600 324		M. McGavin

Supplementary Table 2 - Liquid chromatography-mass spectrometry reveals potential candidates as the inhibitory compound in S. chromogenes supernatant

Name/Formula	Score ratio
Tioguanine	2.26
Acetophenone	2.29
Indoleacrylic acid	2.67
5-Methoxy-3-indoleaceate	3.64
leucoline	5.12
Cinnamic acid	9.11

Name/Formula	Score ratio
C14 H25 N3 O5	1.08
Tioguanine	1.37
N-Acetyl-L-leucine	1.94
Ethopabate	2.13
Oxagrelate	2.28
N-Acetyl-L-phenylalanine	2.63
Cinnamic acid	2.70
Tetraacetylethylenediamine	3.09
Indoleacrylic acid	4.33
Acetylcarnitine	5.89
5-Methoxy-3-indoleaceate	8.84

Name/Formula	Score ratio
N-Acetylcystathionine	1.44
Tioguanine	1.49
N-Acetyl-L-phenylalanine	1.43
Tetraacetylethylenediamine	1.50
Ethopabate	1.60
Indoleacrylic acid	2.12
C17 H21 N7 O3	2.19
Cinnamic acid	2.44
C14 H25 N3 O5	2.66

Mass spectrometry was performed on active fractions from HPLC in three independent experiments. Hits that were present in all three experiments with a score ratio of < 10 are displayed.

Supplementary Table 3 – Primers used in this study.

Name	Sequence	Description
ATCC43674_tgs_F_kpnI	TTTTTTGGTACCCCGAACTTTTATTCGAAAAT	Forward primer to amplify *tgs* operon from *S. chromogenes* ATCC43764
ATCC43674_tgs_R_SacI	TTTTTTGAGCTCCTAGT TTTTAATTGTAAAAC	Reverse primer to amplify *tgs* operon from *S. chromogenes* ATCC43764
VCU116_tgs_F_kpnI	TTTTTTGGTACCTGTTTCTTTTACTTGT CTGC	Forward primer to amplify *tgs* operon from *S. capitis* VCU116
VCU116_tgs_F_SacI	TTTGAGCTCGGTTATTTTCAGTACACTCC	Reverse primer to amplify *tgs* operon from *S. capitis* VCU116

Supplementary References:

1. Goncheva, M. I., Flannagan, R. S. & Heinrichs, D. E. De novo purine biosynthesis is required for intracellular growth of *Staphylococcus aureus* and for the hypervirulence phenotype of a purR mutant. *Infect. Immun.* (2020). doi:10.1128/IAI.00104-20
2. Tse, H. *et al.* Complete genome sequence of *Staphylococcus lugdunensis* strain HKU09-01. *J. Bacteriol.* **192,** 1471–1472 (2010).
3. Heilbronner, S. *et al.* Genome sequence of *Staphylococcus lugdunensis* N920143 allows identification of putative colonization and virulence factors. *FEMS Microbiol. Lett.* **322,** 60–67 (2011).
