# Supplementary material for: Coagulase-negative staphylococci release a purine analog that inhibits Staphylococcus aureus virulence
Source: Nat Commun. 2021 Mar 25;12:1887. doi: 10.1038/s41467-021-22175-3 (PMC7994395; doi:10.1038/s41467-021-22175-3)
Supplement: Supplementary file 2 — Description of Additional Supplementary Files [file 41467_2021_22175_MOESM2_ESM.pdf]

Description of Additional Supplementary Files

File Name: Supplementary Data 1
Description: A list of publicly available NCBI genomes from *S. capitis, S. chromogenes, S. epidermidis and S. pseudintermedius* used in the phylogenetic analyses in Fig 2.

File Name: Supplementary Data 2
Description: RNA-seq analyses of wild-type *S. aureus* and *S. aureus purK*::ΦΝΣ cultures treated with a vehicle control or 6-TG (10 µg/mL) in TSB. Expression values for each transcript were compared between different conditions.
