# Supplementary material for: Coagulase-negative staphylococci release a purine analog that inhibits Staphylococcus aureus virulence
Source: Nat Commun. 2021 Mar 25;12:1887. doi: 10.1038/s41467-021-22175-3 (PMC7994395; doi:10.1038/s41467-021-22175-3)
Supplement: Supplementary file 5 — Reporting Summary [file 41467_2021_22175_MOESM5_ESM.pdf]

nature research

Corresponding author(s): D. E. Heinrichs

Last updated by author(s): February 22, 2021

Reporting Summary

Nature Research wishes to improve the reproducibility of the work that we publish. This form provides structure for consistency and transparency in reporting. For further information on Nature Research policies, see our Editorial Policies and the Editorial Policy Checklist.

Statistics

For all statistical analyses, confirm that the following items are present in the figure legend, table legend, main text, or Methods section.

n/a	Confirmed	
☐	☒	The exact sample size (*n*) for each experimental group/condition, given as a discrete number and unit of measurement
☐	☒	A statement on whether measurements were taken from distinct samples or whether the same sample was measured repeatedly
☐	☒	The statistical test(s) used AND whether they are one- or two-sided<br>*Only common tests should be described solely by name; describe more complex techniques in the Methods section.*
☐	☒	A description of all covariates tested
☒	☐	A description of any assumptions or corrections, such as tests of normality and adjustment for multiple comparisons
☐	☒	A full description of the statistical parameters including central tendency (e.g. means) or other basic estimates (e.g. regression coefficient) AND variation (e.g. standard deviation) or associated estimates of uncertainty (e.g. confidence intervals)
☒	☐	For null hypothesis testing, the test statistic (e.g. *F*, *t*, *r*) with confidence intervals, effect sizes, degrees of freedom and *P* value noted<br>*Give P values as exact values whenever suitable.*
☒	☐	For Bayesian analysis, information on the choice of priors and Markov chain Monte Carlo settings
☒	☐	For hierarchical and complex designs, identification of the appropriate level for tests and full reporting of outcomes
☒	☐	Estimates of effect sizes (e.g. Cohen's *d*, Pearson's *r*), indicating how they were calculated

Our web collection on statistics for biologists contains articles on many of the points above.

Software and code

Policy information about availability of computer code

Data collection	No software used
Data analysis	Graphpad version 7.0 was used for graphical and statistical analyses. ImageJ 1.53g was used for analysis of lesion size in mice. Geneious Prime version 2020.1.1 was used to analyze RNAseq data and genomic data for comparative SNP analysis. For phylogenetic tree building, the RAxML plugin in Geneious Prime was used and the tree imported into MEGA7. DNA sequence reads were trimmed using Trimmomatic version 0.36 and SPAdes version 3.13 was used for read assembly. Contigs were annotated using Prokka version 1.12

For manuscripts utilizing custom algorithms or software that are central to the research but not yet described in published literature, software must be made available to editors and reviewers. We strongly encourage code deposition in a community repository (e.g. GitHub). See the Nature Research guidelines for submitting code & software for further information.

Data

Policy information about availability of data

All manuscripts must include a data availability statement. This statement should provide the following information, where applicable:

- Accession codes, unique identifiers, or web links for publicly available datasets
- A list of figures that have associated raw data
- A description of any restrictions on data availability

The raw sequence reads for the S. chromogenes ATCC43764 (H278Tor, H278Pit, H279), JP98 and JP383 isolates sequenced in this study can be found in the NCBI Sequence Read Archive under BioProject ID: PRJNA630769 (https://www.ncbi.nlm.nih.gov/bioproject/?term=PRJNA630769). RNAseq data can be found in the NCBI Sequence Read Archive under BioProject ID: PRJNA695221 (https://www.ncbi.nlm.nih.gov/bioproject/PRJNA695221). The authors declare that the data supporting the findings of this study are available within the paper and its supporting supplementary information files.

Field-specific reporting

Please select the one below that is the best fit for your research. If you are not sure, read the appropriate sections before making your selection.

- [x] Life sciences
- [ ] Behavioural & social sciences
- [ ] Ecological, evolutionary & environmental sciences

For a reference copy of the document with all sections, see nature.com/documents/nr-reporting-summary-flat.pdf

Life sciences study design

All studies must disclose on these points even when the disclosure is negative.

Sample size	No statistical method was used for the predetermination of sample sizes. Sample sizes primarily pertained to animal studies and varied between experiments and were selected based on our previously published studies and based on the minimum sample size to yield statistical difference from those studies.
Data exclusions	No data were excluded
Replication	All information on replication and experimental repeats can be found in the figure legends. Where murine experiments were repeated, the trend was not different between experiments. Some in vitro experiments in the supplementary figures were done in biological triplicate but performed in only one independent experiment. These data had no effect on any conclusions drawn from the study and thus were not repeated. For the data shown throughout the manuscript, all attempts at repetition were successful.
Randomization	No experiments were performed using clinical subjects therefore randomization does not apply. For our animal studies all mice were randomized into each treatment cohort.
Blinding	Investigators were not blinded to the study, as experimental parameters were bacterial burden and lesion size, and not subjective measures of behavior. Euthanasia of animals was performed according to strict guidelines, as outlined in our animal use protocol.

Reporting for specific materials, systems and methods

We require information from authors about some types of materials, experimental systems and methods used in many studies. Here, indicate whether each material, system or method listed is relevant to your study. If you are not sure if a list item applies to your research, read the appropriate section before selecting a response.

Materials & experimental systems

n/a	Involved in the study	
☐	☒	Antibodies
☒	☐	Eukaryotic cell lines
☒	☐	Palaeontology and archaeology
☐	☒	Animals and other organisms
☒	☐	Human research participants
☒	☐	Clinical data
☒	☐	Dual use research of concern

Methods

n/a	Involved in the study	
☒	☐	ChIP-seq
☒	☐	Flow cytometry
☒	☐	MRI-based neuroimaging

Antibodies

Antibodies used	1. Rabbit anti-staphylococcal α-Toxin antibody (Sigma-Aldrich, Cat. No. S7531, lot #051M4768 used at 1:500)<br>2. Donkey anti rabbit DyLight 800 secondary antibody (Rockland, Cat No 611-745-127, lot #38422,used at 1:20,000).
Validation	1. Technical data sheet "The antiserum has been treated to remove lipoproteins. Rabbit anti-staphylococcal α-toxin antibody reacts specifically with staphylococcal α-toxin but not with staphylococcal enterotoxin A, cholera toxin or pseudomonas exotoxin A."<br>2. Technical data sheet "This product was prepared from monospecific antiserum by immunoaffinity chromatography using Rabbit IgG<br>coupled to agarose beads followed by solid phase adsorption(s) to remove any unwanted reactivities. Assay by immunoelectrophoresis resulted in a single precipitin arc against anti-Donkey Serum, Rabbit IgG and Rabbit Serum. No reaction was observed against Bovine, Chicken, Goat, Guinea Pig, Hamster, Horse, Human, Mouse, Rat and Sheep Serum Proteins. This antibody will react with heavy chains of rabbit IgG and with light chains of most rabbit immunoglobulins."

Animals and other organisms

Policy information about studies involving animals; ARRIVE guidelines recommended for reporting animal research

Laboratory animals	Female BALB/C mice, received from Charles River Laboratories at 6-8 weeks upon arrival.
Wild animals	Study did not involve wild animals
Field-collected samples	Study did not involve samples collected in the field
Ethics oversight	All animal protocols (protocol 2017-028) were reviewed and approved by the University of Western Ontario Animal Use Subcommittee, a subcommittee of the University Council on Animal Care.

Note that full information on the approval of the study protocol must also be provided in the manuscript.
